# Cathepsin Inhibitor Suppresses the Growth of Ectopic Hepatocellular Carcinoma Tumors in Mouse Models

**DOI:** 10.1021/acsptsci.5c00675

**Published:** 2026-03-16

**Authors:** Olamide Crown, Oluwatoyin V. Odubanjo, Olawale S. Adeyinka, Felicite K. Noubissi, Ifedayo Victor Ogungbe

**Affiliations:** † Chemistry and Biotechnology Science and Engineering Programs, 14843 The University of Alabama in Huntsville, 301 Sparkman Drive, Huntsville, Alabama 35899, United States; ‡ Department of Biology, 4114 Jackson State University, Jackson, Mississippi 35217, United States

**Keywords:** hepatocellular carcinoma, cathepsin L, cathepsin S, tumor xenograft, covalent inhibitors

## Abstract

The advanced stages of hepatocellular carcinoma (HCC) are challenging to treat because of the invasion of blood vessels by malignant cells, as well as underlying cirrhosis in most patients. Current chemotherapeutic approaches are hindered by limited efficacy, systemic toxicity, and drug resistance. Therefore, developing new chemotherapeutic agents that can be used alone or as part of a cocktail regimen could aid in the treatment of HCC patients. This study primarily focuses on a cathepsin inhibitor and its antiproliferative effects on HCC cell lines *in vitro* and in HCC tumor xenografts in mouse models. The results show that the inhibitor has significant antiproliferative effects on the Hep G2 and Hep 3B cell lines, with low micromolar CC_50_ values. Furthermore, the compound inhibits both recombinant and endogenous cathepsin L and cathepsin S in a time-dependent manner. In mice, significant reductions in the growth of subcutaneous tumors relative to controls were observed, and it is well-tolerated when compared to Doxorubicin and Sorafenib. Transcriptomics analysis using RNA-Seq revealed that genes involved in regulating cell death, cell proliferation, and cellular processes were enriched in a time- and dose-dependent manner. Overall, the cathepsin inhibitor appears to be a promising starting point for further investigation as an antiproliferative agent.

## Graphical abstract

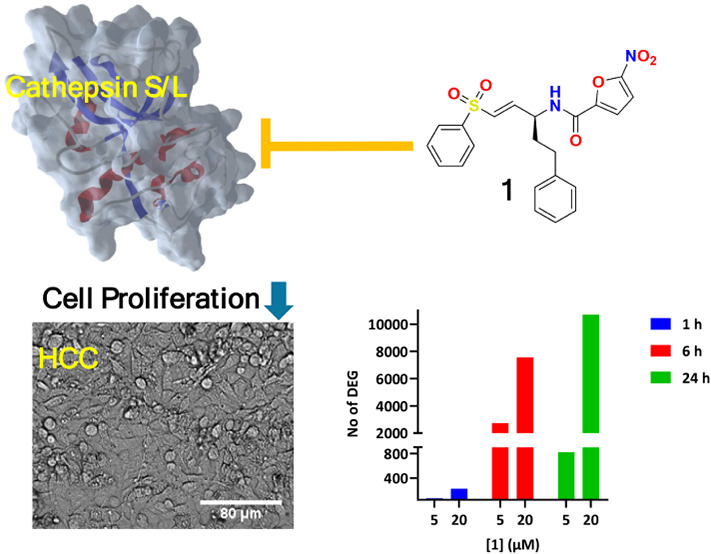

Despite recent advances in treatment, hepatocellular carcinoma is the most prevalent and lethal form of liver cancer often arising from chronic hepatitis B and C viral infections, chronic exposure to aflatoxins, and fatty liver disease.^1,2^ Late-stage diagnoses are frequently associated with poor prognosis, contributing to the high lethality of advanced-stage liver cancer. Chemotherapy is crucial treatment option for advanced hepatocellular carcinoma (HCC), particularly for patients ineligible for surgical resection, local ablative therapy, or trans-arterial chemoembolization (TACE) due to factors like extrahepatic metastasis, vascular invasion, or TACE refractoriness.^3,4^ However, the efficacy of current chemotherapy is still unsatisfactory, and the poor prognosis associated with HCC persists.^5,6^ This is partly due to ongoing and persistent challenges of drug resistance and dose-limiting toxicity.^6,7^ Neither standard chemotherapy, immunotherapy, nor current ablation therapies have significantly improved patient outcomes from advanced HCC.^8^

As a part of our research focus on cysteine protease inhibitors as therapeutic agents, we discovered that **NN9** (compound **1**) has antiproliferative effects on HCC cell lines,^9,10^ and subsequent experiments show that it is weak inhibitor of human cathepsin L inhibitor but a potent inhibitor of cathepsin S. Cathepsin L is a cysteine protease overexpressed in HCC and associated with advanced clinical staging, histological grade, tumor recurrence, tumor aggression, metastasis, and poor prognosis.^7,11–14^ Furthermore, because of its abundance in HCC, it has been proposed as a diagnostic marker as well as a pharmacological target for HCC therapies.^15,16^ Moreover, CatL upregulation has also been found in aggressive cancers such as pancreatic cancer and colorectal adenocarcinoma.^7,16^ A few small molecule inhibitors of cathepsin L have demonstrated the ability to inhibit neoplastic cells and prevent the emergence of drug resistance.^17,18^ Studies have shown that CatL inhibition improved the efficacy of some chemotherapeutic agents by lowering the effective concentrations at which they mediate cytotoxic effects and effectively reverse resistance to a variety of cytotoxic and targeted agents.^19,20^ Similarly, targeted siRNA-mediated knockdown of Cat S expression has been shown to suppress HCC cell proliferation, invasion and angiogenesis.^21,22^ In this article, we report that Compound **1** exhibits promising antiproliferative activity against HCC cell lines *in vitro* and *in vivo*, inhibits both recombinant and endogenous CatL and CatS, and upregulates the expression of genes involved in autophagy and apoptosis.

## Materials and Methods

### Antiproliferation Assay

Hepatocellular carcinoma cell lines Hep G2 (CRL-11997) and Hep 3B2.1–7 (ATCC-HB-8064) were used for the antiproliferation assays. The cells were grown in Dulbecco’s Modified Eagle’s Medium (DMEM)/F12 Ham (Sigma Life Science) containing l-glutamine and sodium bicarbonate, 10% FBS, and 1% penicillin/streptomycin and incubated at 37 °C in a 5% CO_2_ environment. Once the cells were 80–90% confluent, they were washed with PBS, treated with 0.25% (w/v) trypsin/EDTA, counted, and resuspended in fresh complete media. The cells were seeded in 100 μL of the complete medium at a density of 5 × 10^5^ or 2 × 10^5^ cells/mL into 96-well plates and incubated for 24 h for the cells to attach. The cells were treated with compound **1** or its analog **2**, prepared in DMSO at a dose range between 300–0.15 μM for 24, 48, or 72 h in triplicate. Both compounds were synthesized and characterized as previously reported (Scheme S1).^10^ After each incubation period, the medium was removed, and the cells were incubated for 30 min in fresh, serum-free DMEM/F12 medium containing 20% MTT (prepared as 5 mg/mL in PBS). The MTT medium was carefully removed and replaced with DMSO (200 μL/well) to facilitate cell lysis. The lysed cells were repeatedly resuspended in the DMSO medium using a multichannel pipet to aid in the dissolution of the resulting formazan crystals. After incubating the plates for 10 min, the absorbance in each assay well was measured at 550 nm. The assay was carried out in three replicates. Sodium dodecyl sulfate (10%) and podophyllotoxin were positive controls in the assay. The absorbance values in the test wells were expressed as a percentage of absorbance values in DMSO-treated cells to obtain the percentage of cell viability. CC_50_ values were obtained by nonlinear regression curve fit of the mean values using the variable slope model in GraphPad Prism 10.3.1.

### Wound Healing Assay

Hep G2 cells were seeded at a density of 5 × 10^5^ cells/mL in ibiTreat microdish (*Culture-Insert 2 Well in μ-Dish 35 mm*, ibidi USA, Inc.). Aliquots (70 μL) of the cell suspension were placed in half-wells, separated by silicon inserts in the μ-Dish, and incubated for 1 day in a 5% CO_2_ environment at 37 °C to enable proper cell attachment. The silicon inserts were removed, leaving a “wound gap” of 500 ± 50 μm between the attached HepG2 cells. The cells in different dishes were treated with 7.5 or 10 μM of compound **1** or 1% DMSO (assay vehicle) and incubated at 37 °C in a 5% CO_2_ environment. The closure of the wounds was then monitored for 96 h. Images were captured with an Olympus CKX41 microscope at 4× magnification.

### Cathepsin B, L, and S Inhibition Assays

#### Inhibition of Recombinant Cathepsin L and B

The proteolytic activities of recombinant human cathepsin L and B (Biovision Inc., USA) were monitored using fluorescent peptide substrates Z-Phe-Arg-AMC and Z-Arg-Arg-AMC, prepared as 10 mM stock solutions in methanol, respectively. The inhibition assays were performed in a 50 mM sodium acetate buffer at pH 5.5 containing 50 μM DTT, 4 mM EDTA, and 0.01% Triton X-100. For the IC_50_ assays, the enzyme–inhibitor reactions (100 μL) were incubated for 1 h before the substrates (20 μM, 100 μL) were added. For the time-dependent assays, the reaction mixtures include 15 μL of the inhibitors in DMSO, 15 μL of recombinant cathepsin L (0.58 nM) or B (2 nM) in assay buffer, and 260 μL of assay buffer. At 10 min intervals, 40 μL aliquots of the cathepsin-compound mixtures were assayed with 40 μL of the substrate (20 μM). The fluorescence (RFU/sec) resulting from proteolytic cleavage of the substrate was measured (λ_ex_ = 355 nm and λ_em_ = 460 nm) on a PolarStar Omega plate reader (BMG LABTECH, Germany) for 9 min at 25 °C. E-64d (10 μM) was used as the positive control (100% inhibition). The assays were performed in triplicate. The reaction rates were calculated using GraphPad Prism version 10.3.1 and used to obtain the second-order inactivation constant *k*_inact_/*k*_i_ with the one-site tight binding model *k*_obs_ = (*k*_inact_ * [I])/([I] + *k*_i_).

#### Inhibition of Recombinant Cathepsin S

The proteolytic activities of recombinant human cathepsin S (Millipore Sigma, USA) were monitored using fluorescent peptide substrates Z-VVR-AMC, prepared as 10 mM stock solutions in methanol. The inhibition assays were performed in a 35 mM sodium acetate and 35 mM potassium phosphate buffer at pH 6.5, containing 2 mM DTT, 2 mM EDTA, and 0.1% Brij. E-64d (10 μM) and LHVS (10 μM) were used as positive controls (100% inhibition). The IC_50_ assays were performed as described above. For the time-dependent assays, 5 μL of the inhibitors in DMSO, 5 μL of recombinant cathepsin S (1 nM) in assay buffer, 90 μL of assay buffer, and substrate (20 μM, 100 μL) were added in assay plates, and the reaction progress was monitored for 2 h as described above. The reaction rates at 40 min intervals were used to obtain *k*_obs_. *k*_inact_/*k*_i_ values were obtained as described above.

#### Inhibition of Endogenous Cathepsin B/L/S in Lysates from Hep G2 Cells

**Cell Lysis**: Hep G2 cells were lysed in chilled lysis buffer (100 mM potassium phosphate (pH 6.0) containing 100 mM NaCl and 0.1% Triton X-100) for 10 min. The lysate was centrifuged for 14 min at 12,000 rpm in a microcentrifuge, and the supernatants were transferred to a new tube. Aliquots were kept at −80 °C until analyzed. The Pierce 660 nm Protein Assay Reagent was used to determine protein concentrations, which were then normalized to 1 mg protein/mL (CatB/L) or 200 μg protein/mL (CatS) in the lysis buffer. **Assay**: For the inhibition studies, 50 μL of compound **1** (0.1–50 μM) in DMSO, 100 μL of cell lysate in assay buffer, and 850 μL of assay buffer were incubated for 2, 4, 6, or 24 h. At each time point, 100 μL aliquots of the lysate-compound mixtures were assayed in black 96-well plates with 100 μL of Z-FR-AMC (CatL), Z-RR-AMC (CatB), or Z-VVR-AMC (CatS) as substrate (20 μM) was added to each sample, and the fluorescence was measured for 20 min as described above.

#### Inhibition of Endogenous Cathepsin B/L/S in Lysates by Pretreatment of Live Hep G2 Cells

**Cell Treatment**: Hep G2 cells (2.5 × 10^6^ cells/mL) were seeded in 6-well plates and incubated at 37 °C in 5% CO_2_ overnight. The cells were treated with **1** (0.1–50 μM) and incubated for 2, 4, 6, or 24 h. After incubation, the medium was removed, and the cells were rinsed with chilled PBS and lysed as described above. **Assay**: 10 μl of the lysates (1 mg protein/mL) for CatB/L assay or 5 μL (200 μg protein/mL) for CatS assay were aliquoted into black 96-well plates, 90 μL of assay buffer was added, 100 μL of 20 μM substrate was added, and the fluorescence was measured as described above.

#### Magic Red Cathepsin Activity Detection Assay

Hep G2 cells (3 × 10^5^ cells/mL) were seeded into 6-well plates in phenol red-free medium containing 2% FBS and incubated at 37 °C in 5% CO_2_ overnight. The medium was removed from the attached cells and replaced with fresh medium, and the cells were treated with 5 and 10 μM of compound **1** for 6 h. The cells were rinsed with PBS and incubated with the Magic Red dye (diluted 50-fold) for cathepsin L or cathepsin B (ImmunoChemistry Technologies, Davis, CA) for 45 min in phenol red-free media containing 2% FBS. Subsequently, they were rinsed with PBS, and 500 μL of the medium was added to prevent cell starvation. The fluorescence images were captured (λ_ex_ = 560 nm and λ_em_ = 620 nm) with an Olympus CKX41 microscope controlled by Ocular Version 2.0.

### Quantification of Reactive Oxygen Species (ROS)

#### Microplate Assay

Intracellular ROS levels were evaluated using the fluorescent probe dichlorofluorescein diacetate (H_2_DCFDA, Sigma-Aldrich, MO). Hep G2 or Hep 3B cells were seeded and incubated in black 96-well plates at a density of 50,000 cells/well in phenol red-free media containing 2% FBS.^37^ The cells were pretreated with 50 μM H_2_DCFDA for 30 min and subsequently treated with compound **1**/**2** in DMSO (0.1–50 μM) or H_2_O_2_ (25–250 μM) as positive control or DMSO as vehicle control. The fluorescence signals were measured (λ_ex_ = 485 nm and λ_em_ = 535 nm) at 2.5, 4, 6, 12, 18, or 24 h on a PolarStar Omega plate reader (BMG LABTECH, Cary, NC). Subsequently, the fluorescence signals were normalized to the fold change relative to the DMSO control and presented as heatmaps, as shown in the result section below, using GraphPad Prism 10.3.1. The assays were repeated independently three times.

#### Microscopy Assay

Hep G2 cells (2.5 × 10^5^ cells/well) were seeded into 6-well plates in phenol red-free medium containing 2% FBS. The cells were treated with **1** (1–50 μM) for 4 h, after which the treatment was removed, and the cells were incubated with 20 μM H2DCFDA for 30 min at 37 °C. The cells were then washed three times with fresh phenol-red free media containing 2% FBS, and the images were captured at λ_ex_ = 560 nm and λ_em_ = 620 nm with an Olympus CKX41 microscope controlled by Ocular Version 2.0.

#### Ectopic Xenograft Assay in Nude Mice

Immunocompromised mice (Nude mice; Foxn1^nu^; Jackson Laboratory Stock number 007850) were used for this assay. In individually ventilated cages, the mice were acclimated for 2 weeks and fed γ-irradiated control diets (Bio-Serv, New Jersey, USA). **Tumor Induction and Treatment:** The implantation and treatment were carried out as depicted in Figure 1. Hep 3B2.1–7 cells were grown synchronously in T300 flasks. At 80% confluence, the cells were treated with trypsin, washed with sterile PBS, harvested, and then resuspended in sterile PBS. Aliquots containing 6 × 10^6^ viable cells in 100 μL PBS were transferred into sterile tubes, followed by the addition of 100 μL of chilled Matrigel (Corning, USA). The resulting 1:1 PBS:Matrigel mixtures were maintained on ice to prevent polymerization. The cell suspension (150 μL) was injected subcutaneously into the midleft flank of each mouse using a 27-gauge needle.

**Figure 1:**
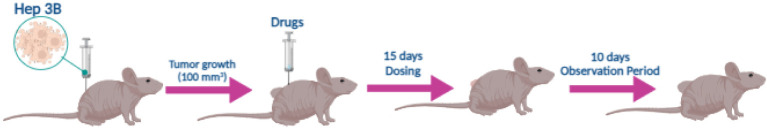
Schematic diagram of the implantation and treatment schedule of the HCC ectopic xenograft in mice.

The mice were randomized into cohorts of 5 animals per group. The groups include vehicle (5% DMSO and 5% PBS in corn oil), 100 mpk compound **1**, 150 mpk compound **1**, positive control doxorubicin (Dox, 7.5 mpk), and positive control Sorafenib (Sor, 30 mpk). Tumor development was monitored until the tumors reached approximately 100 mm^3^. Mice that developed tumors received subcutaneous injections (100 μL) of 100 mpk (n = 5) of **1**, 150 mpk (n = 5) of **1,** or vehicle on the left flank, close to the tumor once per day for 15 days, doxorubicin (Dox) group (n = 5) were injected subcutaneously at 7.5 mpk daily for 7 days (treatment was terminated after 7 days due to signs of systemic toxicity), while 30 mpk of Sorafenib (n = 5) was administered orally. The 7.5 mpk 7-day short-course subcutaneous dosing with doxorubicin was expected to be effective in suppressing tumor progression. Oral dosing with Sorafenib at 30 mpk was expected to effectively reduce tumor progression with known systemic toxicity, as previously shown.^7^ The subcutaneous route for compound 1 was selected because of the rapid clearance observed when administered at 1 mpk (i.v), with a clearance rate of 201 ± 60.6 mL/min/kg and a half-life of 17 min in a pharmacokinetics assay. Tumors were measured before treatment started (day 0) and on days 7 and 14. The animals were observed for 10 additional days (post-treatment) to monitor the effects of the compounds’ withdrawal on tumor progression. There was no masking during treatment.

Mice were euthanized via CO_2_ asphyxiation. Blood samples were collected through cardiac puncture into heparinized bottles, spun at 3000*g* for 10 min, and the plasma samples were stored at −80 °C before analysis. The tumors were harvested, measured, and fixed with formalin. The fold change in tumor volumes at the end of the treatment relative to the start was determined. The data are presented as mean fold change ± standard deviation (X ± SD) and analyzed with ANOVA and Tukey-Kramer posthoc test. A two-tailed *p*-value <0.05 was regarded as statistically significant. The plasma levels of aspartate transaminase, alanine transaminase, blood urea nitrogen, and creatinine were analyzed using a VET AXCEL Chemistry Analyzer. All procedures and predeath monitoring criteria were approved (Protocol No. 20–02) by the Jackson State University’s Institutional Animal Care and Use Committee.

## Transcriptomics Analysis

### Cell Treatment

Hep G2 cells (1 × 10^6^ cells/well) were seeded in 6-well plates and treated with 5 or 20 μM of compound **1** or DMSO (vehicle) for 1, 6, or 24 h in triplicate (Figure 2). The cells were harvested, rinsed with PBS, and then preserved at −80 °C.

**Figure 2:**
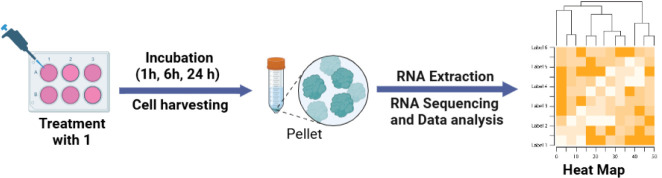
Summary of transcriptomics analysis of HepG2 cells treated with Compound **1**.

### RNA Isolation and Bulk RNA Sequencing

RNA samples were extracted from the cells using TRIzol Reagent along with Pure Link RNA Mini Kit (Invitrogen) according to manufacturer instructions and assessed for quality control parameters of minimum concentration and fidelity (i.e., 18S and 28S bands, RIS > 7). Libraries were developed using the Illumina TruSeq mRNA Stranded Library Prep Kit (IDT for Illumina – TruSeq RNA UD Indexes), quantified with a Qubit fluorimeter (Invitrogen), and assessed for quality and size using the QIAxcel Advanced System as previously described (Dungan et al., 2021; Johnson et al., 2020). Samples were pooled into a single library (n = 27 pooled samples per library) and sequenced using the NextSeq 2000 P3 200 cycles (paired-end 100 bp) on the Illumina NextSeq2000 platform. The run generated approximately 1.2 billion reads (%QC30 = 93) or 26 million reads passing the filter per sample. Sequenced reads were assessed for quality using the Illumina BaseSpace Cloud Computing Platform, and FASTQ sequence files were used to align reads using the STAR aligner (v2.7.10) to the human reference genome (GRCh38, Ensembl release 108) with corresponding annotation GTF files. On average, >96% of reads per sample are mapped to the Human genome. Differential expression was determined using the RNA-Differential Expression Application (v1.0.1, 4/22/21) and DESeq2. Gene expression differences are denoted as log_2_ (ratio) and *q* > 0.05.

### Read Alignment and Transcript Quantification

The raw reads were aligned using the STAR aligner (v2.7.10) to the human reference genome (GRCh38, Ensembl release 108) with corresponding annotation GTF files. The alignment was performed with default parameters, and uniquely mapped reads were retained for downstream analysis. To estimate transcript-level abundances, the raw reads were also processed using Salmon (v1.8.0). The transcriptome index was constructed using the above reference and the corresponding annotation GTF file. The quantification was conducted in mapping-based mode with default settings, generating transcript-level abundance estimates.

### Differential Expression Analysis

The differential expression analysis was conducted using the DESeq2 package in R (v4.2.0). The gene-level count data obtained from Salmon were used for this analysis. The raw count data were imported into R using TX import 1.20 to aggregate the counts across samples to acquire the summarized gene counts for the analysis, and lowly expressed genes (defined as those with low counts across samples) were filtered out. The remaining count data were then normalized using the DESeq2 package’s built-in normalization methods, accounting for differences in library sizes and biological variability. The normalized count data were used to perform differential expression analysis. A negative binomial distribution model was applied to model the count data, and dispersion estimation was performed for each gene. Differential expression analysis was carried out by fitting the model and considering the experimental design and conditions of interest. The samples were also compared over a time series. To control for false positives due to multiple hypothesis testing, the resulting p-values were adjusted for multiple testing using methods such as the Benjamini-Hochberg procedure. Genes with an adjusted p-value below 0.05 (*p* < 0.05) were considered statistically significant. Volcano plots, MA-plots, heatmaps, and principal components analysis plots based on “regularized log” transformation were used to visualize the results of the differential expression analysis and were generated with R packages ggplot2, Enhanced Volcano plot, cluster, and heatmap. These visualizations aided in highlighting the most significantly upregulated and downregulated genes and patterns across the treatment conditions.

### Analysis of Candidate Genes and Cellular Pathways

Multiple cross-comparisons were generated and assessed. However, the focus of this study involved grouping variations of transcriptomic profiles across time and compound **1** concentrations. Transcript feature counts were consolidated into counts based on individual gene names.^23^ Group-to-group comparisons generated output base means of sequence read counts, adjusted P-values for group consistency, and overall fold changes relative to the normalized group (as well as some additional statistical information). The total number of genes annotated was 60,000. About 30,000 genes were expressed and tested. The top 30 genes were used for subsequent gene ontology (GO) terms and pathway analysis. Individual candidate cellular pathways were analyzed using the Database for Annotation, Visualization and Integrated Discovery (DAVID version 6.8; https://david.ncifcrf.gov/, Kyoto Encyclopedia of Genes and Genomes (KEGG) pathway database reports.^24–26^ The interconnectedness of cellular pathways based upon these genes was further assessed using GOnet (https://tools.dice-database.org/GOnet/) from the DICE-Tools online database (Database of Immune Cell Expression, Expression quantitative trait loci and Epigenomics).^27^ Reported pathway connections were restricted to those with false-discovery-rate (FDR) adjusted p-values <0.05.

### Gene Expression Analysis Using Quantitative Real-Time PCR

Total RNA was extracted from Hep G2 cells treated with 5 μM or 20 μM of compound **1**, with DMSO-treated cells serving as the control, using TRIzol reagent. Cells from two biological replicates per condition, cultured in a six-well plate, were harvested with 1,000 μL of TRIzol reagent, transferred to 1.5 mL microcentrifuge tubes, and incubated at room temperature for 5 min. Subsequently, 250 μL of chloroform was added, and the samples were vortexed and incubated at room temperature for 10 min. Phase separation was achieved by centrifugation at 14,000 rpm for 15 min, after which the aqueous (upper) phase was carefully transferred to a new tube. RNA was precipitated by adding an equal volume of isopropanol, mixing vigorously, incubating at room temperature for 10 min, and then centrifuging at 14,000 rpm for 15 min. The resulting RNA pellet was washed with 70% ethanol, air-dried for approximately 15 min, and resuspended in DEPC-treated water. RNA concentration and purity were determined using a NanoDrop spectrophotometer. Complementary DNA (cDNA) was synthesized using the High-Capacity cDNA Reverse Transcription Kit (Applied Biosystems). Quantitative real-time PCR (qRT-PCR) was performed using a QuantStudio 3 Real-Time PCR System in triplicate. Each reaction contained 10 μL of PowerUp SYBR Green Master Mix, 500 nM each of forward and reverse primers (Table 1), and a 1:10 dilution of the cDNA. All reactions were performed in technical triplicate. Gene expression levels were calculated using the 2-^ΔΔCt^ method, with β-actin as the internal control gene. The data are presented as mean fold change ± standard deviation (X ± SD) and analyzed with ANOVA and Tukey-Kramer posthoc test.

**Table 1:** Specific Primer Information for Validated Genes

Gene name	Forward primer	Reverse primer
**sqstm1**	agaatcagcttctggtccatcg	tccgtgctccacatcgatatc
**gabarapl1**	ggccaactgtatgaggacaatc	gcttccaaccactcatttccc
**akr1c1**	ttggtgcaattcccatcgac	tcctcacctggctttacagac
**angptl8**	cttaaaggctcacgctgacaag	gagtctctcctggatctgtcg
**akap12**	accaagctcctacagaagaatgg	ttcttcctcctggctgtttagg
**actβ**	aaagacctgtacgccaacac	ctcaggaggagcaatgatcttg

### Western Blotting

Hep G2 cells were seeded in T-25 flasks at 4.5 × 10^6^ cells/flask and treated with 5 or 20 μM of compound **1** or DMSO (vehicle) for 6 or 24 h. The cells were rinsed with ice-cold PBS and lysed in ice-cold lysis buffer containing 100 mM potassium phosphate (pH 6.0), 100 mM NaCl, and 0.1% Triton X-100, supplemented with Pierce protease inhibitor cocktail (Thermo Scientific, Waltham, MA). The cell lysates were incubated on ice for 20 min and then centrifuged at 14,000*g* for 10 min at 4 °C. Protein concentrations were determined using the Pierce 660 nm reagent and normalized prior to analysis. Equal amounts of protein (40 μg) were mixed with Laemmli buffer, boiled at 95 °C for 10 min, and resolved on 4–20% gradient SurePAGE, Bis-Tris gels (GenScript) using Tris-MOPS-SDS (GenScript) running buffer. Proteins were transferred onto nitrocellulose membrane (Amersham Protran 0.45 μm) using tris–glycine transfer buffer (pH 8.3) containing 20% methanol at 110 V and 300 mA for 90 min. The membranes were blocked in TBST (0.1% Tween-20) containing 5% nonfat milk for 1 h and incubated overnight at 4 °C with an apoptosis antibody cocktail detecting pro/p17-caspase-3, cleaved PARP-1, and β-actin (Abcam, ab136812; 1:250 dilution). Following TBST washes, the membrane was incubated with cocktail secondary antibodies (HRP-conjugated goat antirabbit and antimouse, 1:100 dilution) for 1 h at room temperature. Protein bands were visualized using enhanced chemiluminescence and imaged with a Bio-Rad ChemiDoc Touch imaging system. Images were acquired under nonsaturating conditions and analyzed using Image Lab software (Bio-Rad v6.1). Band intensities were background-subtracted, quantified as adjusted volume, and normalized to β-actin. Data are presented as percentages relative to the control from a representative blot.

## Results

### Compound 1 and Its Analog Have Antiproliferative Effects on Hepatocellular Carcinoma Cells

Compounds **1** and **2** inhibited the growth of hepatocarcinoma cells (HCC) (Hep G2 and Hep 3B cells) in a time-dependent manner, and both compounds exhibited low micromolar CC_50_ values (Figure 3-IA–D). The antiproliferative effect varied with time (24, 48, and 72 h) and cell population (20,000 and 50,000 cells/well). After 72 h, compound **1** demonstrated greater activity on Hep G2 than **2** (Figure 3-IA), but both compounds have similar effects on Hep 3B. Due to the superior cytotoxic effect of compound **1** on Hep G2, it was tested at 7.5 and 10 μM in a wound healing assay to assess its impact on cell proliferation. The compound inhibited cell proliferation at both concentrations in a time-dependent manner compared to the vehicle-treated cells (Figure 3-II). The vehicle-treated cells were proliferating, and the wound’s diameter was significantly reduced after 96 h. At 7.5 μM (IC_50_), the compound significantly inhibited cell motility at 96 h, whereas at 10 μM, the compound inhibited cell proliferation, and floating cell debris was evident in the culture dish (Figure 3-II).

**Figure 3:**
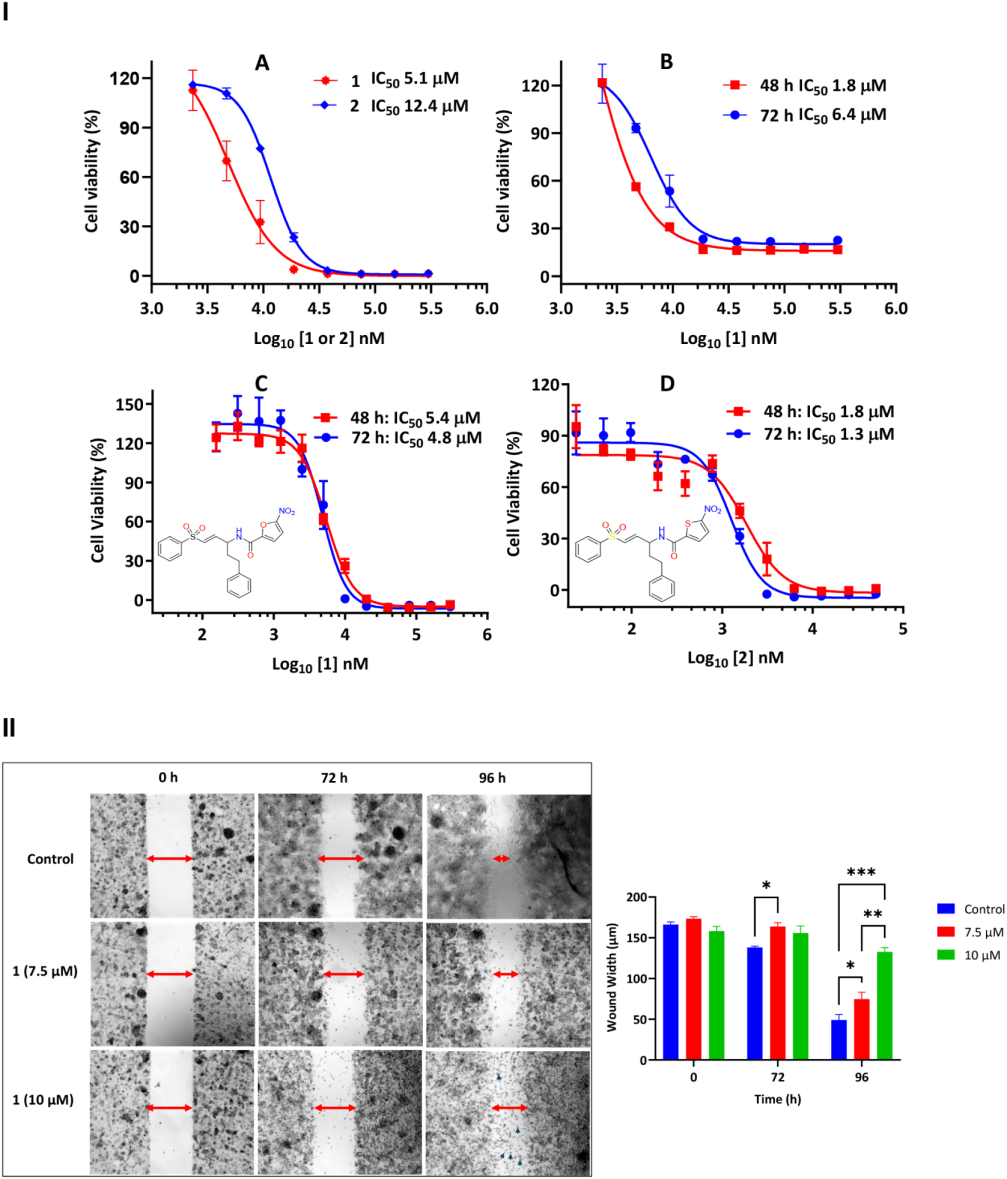
Antiproliferative activity of Compound **1** and its analog, **2** on hepatocellular carcinoma cells. (IA) Hep G2 cells treated with **1** and **2** for 72 h; (1B) Hep G2 cells treated with **1** for 48 and 72 h; (1C) Hep 3B cells treated with **1** for 48 and 72 h; (1D) Hep 3B cells treated with **2** for 48 and 72 h. *n* = 3 in all. (II) Antiproliferative effect of compound **1** in a wound healing assay. The red arrow shows the wound width (the distance between the two margins of the scratch) while the blue arrow depicts the floating and nonviable Hep G2 cells. Wound widths were quantified using ImageJ (NIH). All measurements were reported in micrometers (μm). Data are presented as mean ± SD from independent wounds (*n* = 3). Statistical significance was determined using two-way repeated-measures ANOVA with Geisser–Greenhouse correction, followed by Tukey’s multiple-comparisons test (*p < 0.05, **p < 0.01, ***p < 0.001).

### Compounds 1 and 2 Can Inhibit the Proteolytic Activity of Recombinant Cathepsin S and L as Well as Endogenous Cathepsin S and L in Hep G2 and Hep 3B Cells

Because the compounds were initially designed as covalent inhibitors of the cathepsin L-like protease in *Trypanosoma brucei*, their ability to inhibit human cathepsin L was investigated. Both compounds displayed weak but time-dependent inhibition of recombinant human cathepsin L with IC_50_ values of 6.2 and 3.2 μM, and *k*_inact_/*k*_i_ values of 80 and 223 M^–1^s^–1^ for **1** and **2**, respectively (Figures 4 and S1). Subsequent investigation of their inhibitory activity on human cathepsin S revealed that both compounds are stronger inhibitors of cathepsin S than cathepsin L, with IC_50_ values of 81 and 65 nM, and *k*_inact_/*k*_i_ values of 456 and 2042 M^–1^s^–1^, for **1** and **2**, respectively. However, neither compound could inhibit cathepsin B appreciably, even at concentrations up to 50 μM. Because compound **1** was consistently more active in the antiproliferation assays, its ability to inhibit endogenous cathepsin L and S was further investigated by measuring the residual activities of cathepsin L and S in Hep G2 cells pretreated for 2, 4, 6, and 24 h (Figure 5A,B) and performing inhibition assays using lysates from Hep G2 cells (Figure 5C,D). The results show that the activity of cathepsin L was inhibited in both concentration and time-dependent manner. The inhibitory activity of **1** was further investigated using the Magic Red cathepsin L assay. The assay showed that the emission of red fluorescence (cresyl violet) was markedly less in the cell population treated with 5 and 10 μM of compound **1,** which points to a decrease in cathepsin L activity in those cells relative to control cells (Figure 6).

**Figure 4:**
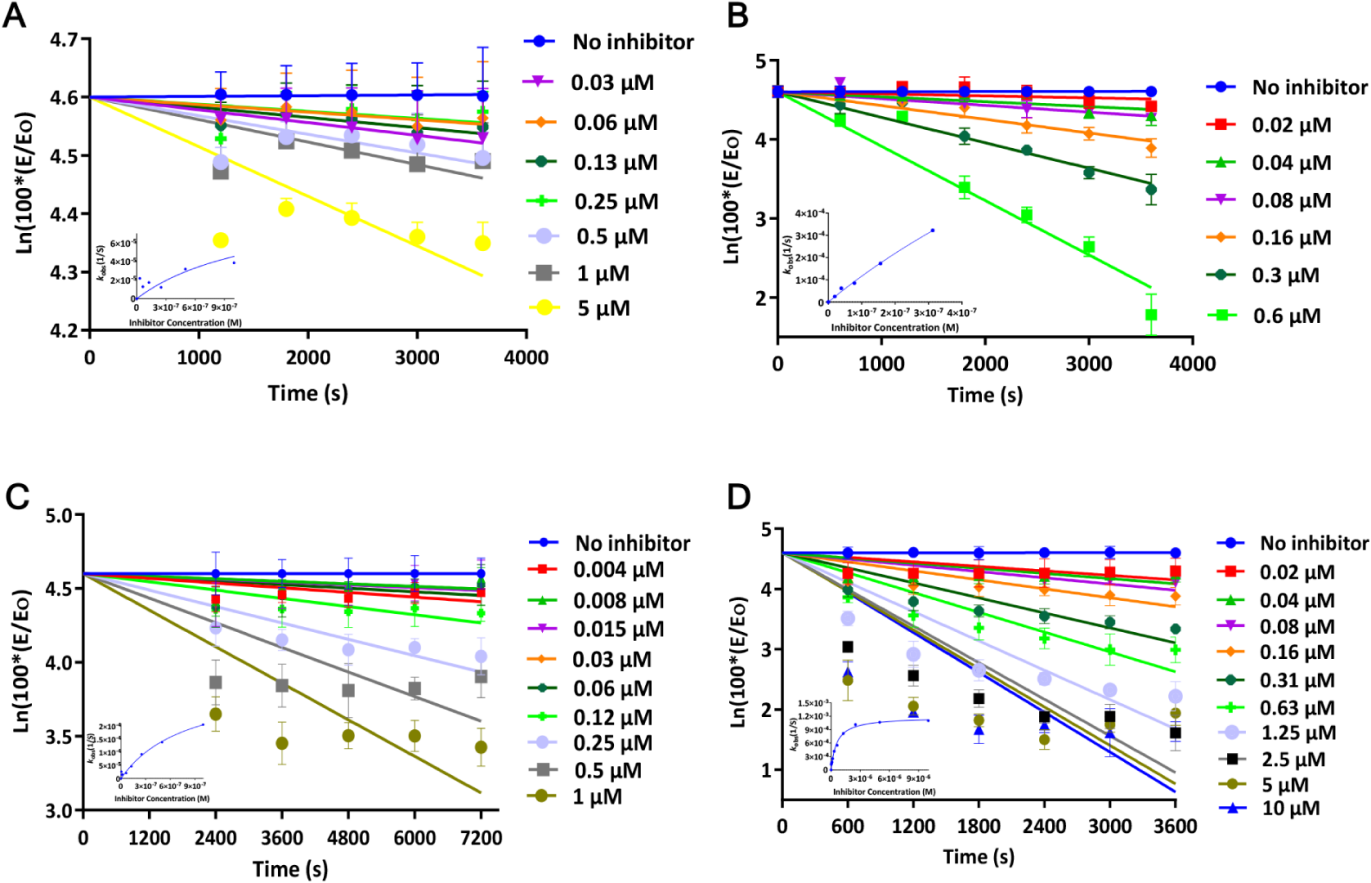
Time-dependent inhibition of cathepsin L (A, B) and S (C, D) by 1 and 2, respectively. The plots show the Ln­(100*E/E_o_) vs time plots which were used to obtain the *k*_obs_ values. The inserts show the second order (*k*_obs_ vs inhibitor concentration) plots to obtain the *k*_inact_/*k*_i_ values. The *k*_inact_/*k*_i_ values for **1** and **2** were 80 and 223 M^–1^s^–1^ for cathepsin L, respectively, while those the values for **1** and **2** were 457 and 2042 M^–1^s^–1^ for cathepsin S, respectively. The second order inactivation constant *k*_inact_/*k*_i_ was obtained using the one-site tight-binding model: *k*_obs_ = (*k*_inact_ * [I])/([I] + *k*_i_). *n* = 3 in all.

**Figure 5:**
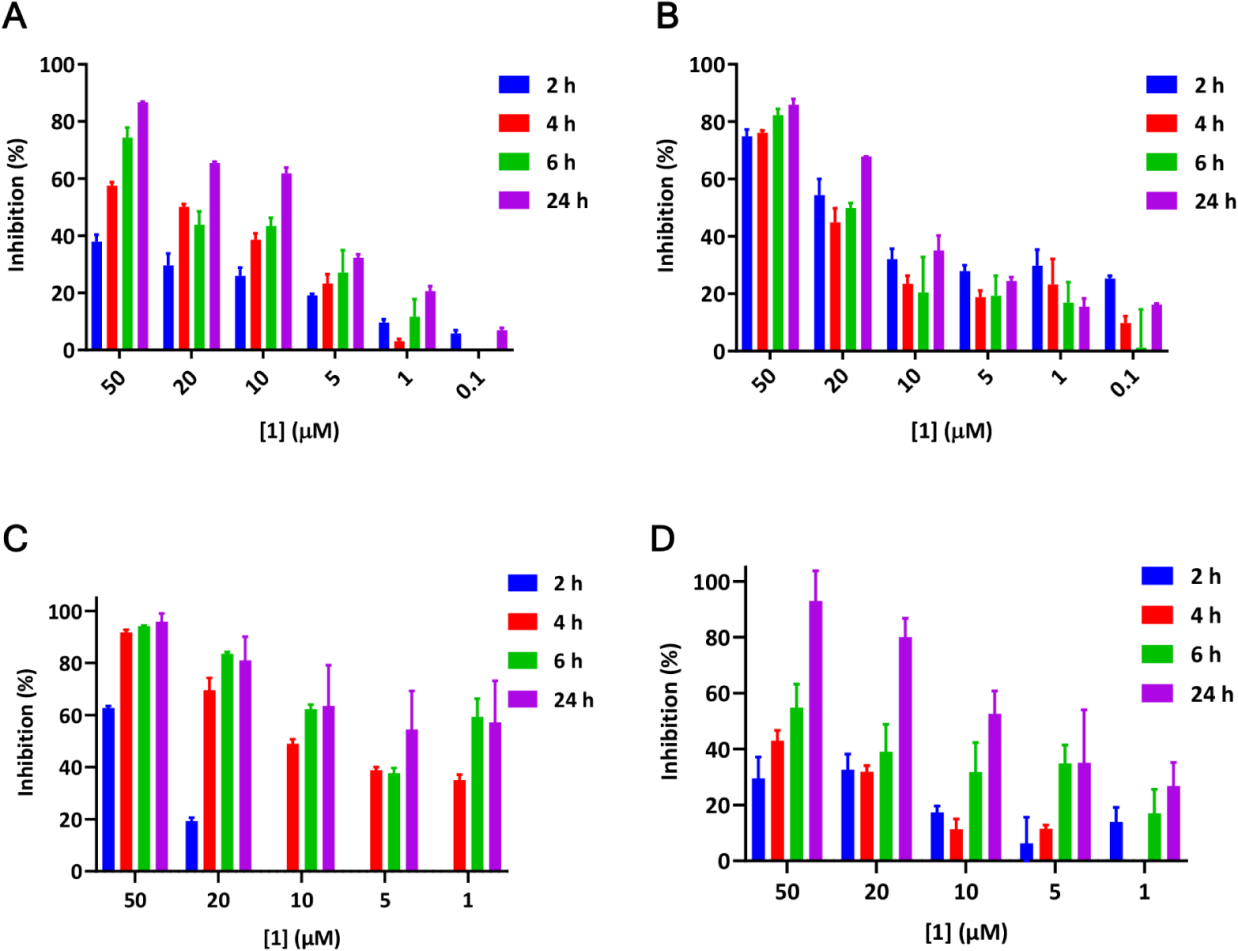
Compound **1** can inhibit cathepsin L and S activities in Hep G2 cells. (A, C) Inhibition of cathepsin L and S activities, respectively, in Hep G2 cells pretreated with **1** for 2, 4, 6, and 24 h relative to control (DMSO). (B, D) Inhibition of endogenous cathepsin L and S activities, respectively, in lysates prepared from frozen Hep G2 cells. *n* = 3 in all.

**Figure 6:**
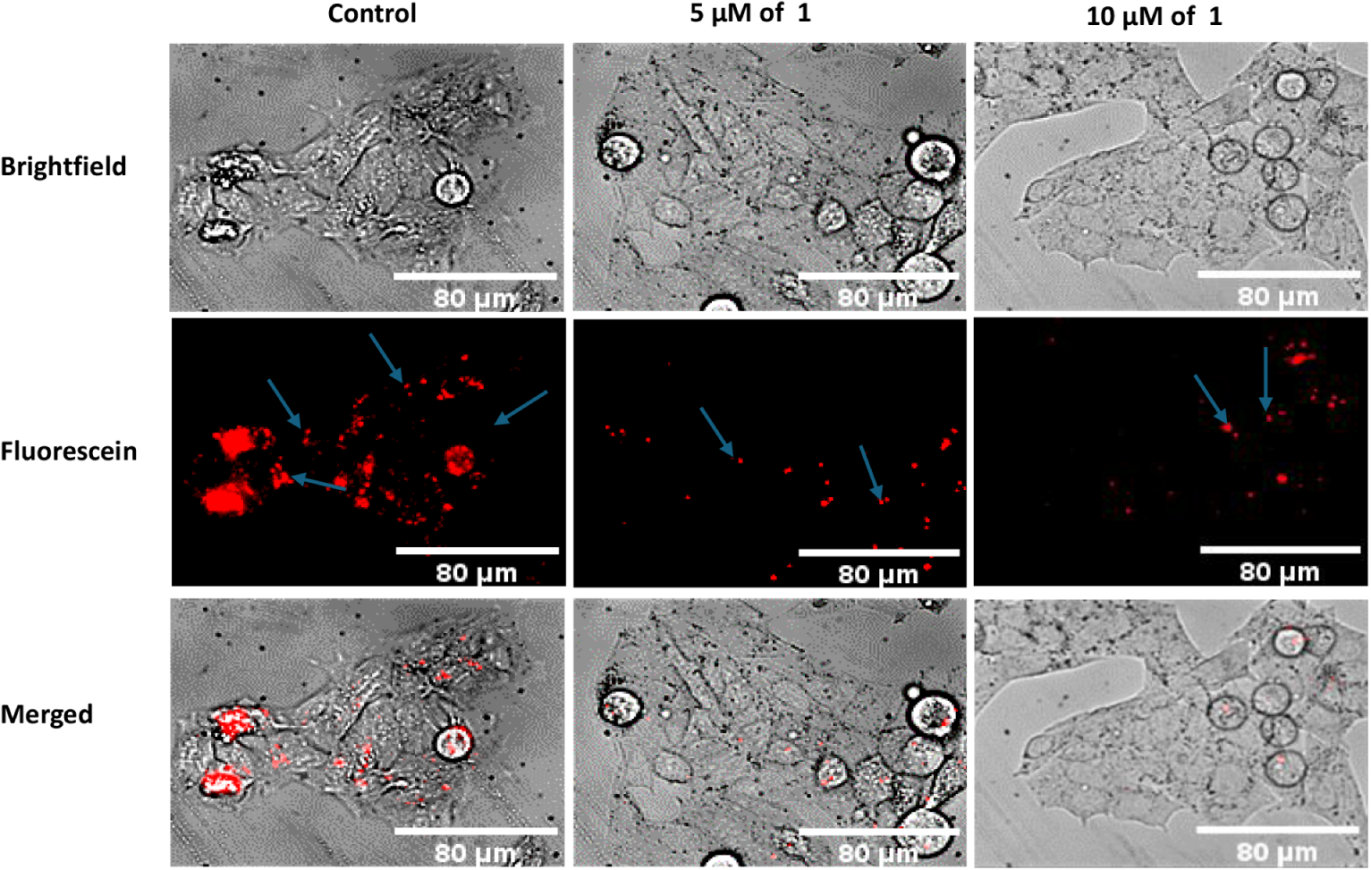
Compound **1** can inhibit endogenous cathepsin L activity in Hep G2 cells. The blue arrows depict areas with intense red fluorescence emitted by cresyl violet when released from the cathepsin L substrate. The hydrolysis of the Magic Red dye (FR)_2_-cresyl violet, fluorescent probe in cells treated with 5 and 10 of **1** μM (center and right) for 6 h was markedly reduced relative to DMSO-treated control (left).

### Compound 1 Does Not Induce the Production of Reactive Oxygen Species at Low but Efficacious Concentrations *In Vitro*

The presence of the nitroaromatic moieties, nitrofuran and nitrothiophene in compounds **1** and **2**, respectively, prompted us to consider the possibility that the compounds are potential substrates for reductive bioactivation, which can result in ROS-mediated cytotoxicity. Therefore, we investigated the levels of intracellular ROS in Hep G2 and Hep 3B cells treated with **1** (0.1–50 μM) relative to DMSO (negative control) and hydrogen peroxide (positive control) using H_2_DCFDA as ROS probe. As shown in the heatmap in Figure 7A,B, there was no major change in the levels of ROS in cells treated with **1 and 2** over a 2.5–24-h period, relative to DMSO, unlike in cells treated with hydrogen peroxide. Cells treated with hydrogen peroxide generated significantly higher levels of ROS both in microplate and microscopy assays. This is evident in the images in Figures 7C, S2, and S3; the intensity of green fluorescence was higher in cells treated with hydrogen peroxide than those treated with **1** and DMSO. In addition, the maximum fluorescent signal from DCF at different concentrations of **1**, **2**, and hydrogen peroxide was at 2.5 h and decreased over time. These results suggest that the antiproliferative effect of **1** is unlikely to be caused by the generation of ROS.

**Figure 7:**
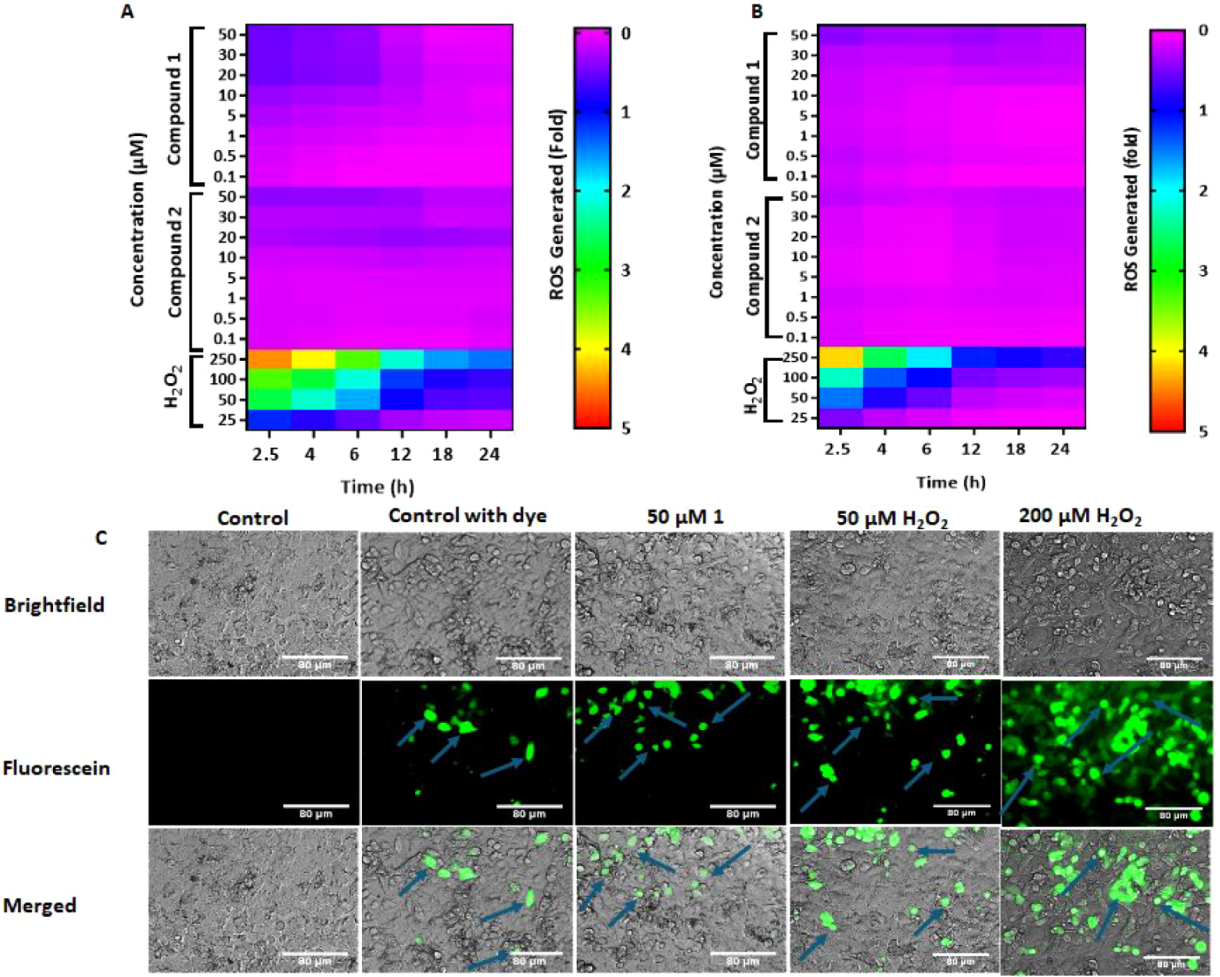
Levels of intracellular reactive oxygen species (ROS) in Hep G2 and Hep 3B cells. (A, B) depict the heatmap of fold change in ROS in Hep G2 and Hep 3B cells treated with compounds **1** and **2**, and hydrogen peroxide relative to DMSO, respectively. The cells were treated for 2.5–24 h at 0.1–50 μM for **1** and **2** and at 25–250 μM for hydrogen peroxide. *n* = 3 for all. (C) Brightfield and fluorescence images of cells treated with **1** and hydrogen peroxide for 6 h. The blue arrows point to the green fluorescence from 2′,7′-dichlorofluorescein (DCF).

### Compound 1 Shows Antiproliferative Activity in Subcutaneous Ectopic Xenografts

Compound **1**’s ability to reduce the growth of tumors in nude mice and the impact it might have on the animals’ livers compared to doxorubicin and Sorafenib was evaluated. It was administered subcutaneously in the left flank and proximal to the tumor for 15 days at 100 or 150 mpk because of its relatively high intrinsic clearance rate (315.7 μL/min/mg) and short half-life (22 min) in mouse liver microsome assay. As shown in Figures 8 and S4, compound **1** significantly attenuated the growth of the tumor xenografts in a dose-dependent manner compared to the vehicle-treated animals (Figure 8A,B). Treatments with compound **1** at 100 mpk and 150 mpk were well tolerated as judged by weight, feeding rates, and fatigue. The plasma levels of aminotransferases (ALT and AST) in animals treated with Sorafenib and doxorubicin were significantly higher than in animals treated with compound **1** or the vehicle (*P* < 0.0001), indicating significantly less impact on the liver (Figure 8C). The creatinine (CREAT) and blood urea nitrogen (BUN) levels, blood biomarkers for kidney function, were not significantly different across the treatment groups (Figure 8D). Additionally, Sorafenib and doxorubicin caused significant weight reduction over the course of treatment (Figure S4). These results revealed that compound **1** has antiproliferative effects on xenograft tumors, albeit with weaker efficacy than Sorafenib and doxorubicin but is more tolerable to mice based on liver biomarkers and gross toxicity assessments at the tested doses.

**Figure 8:**
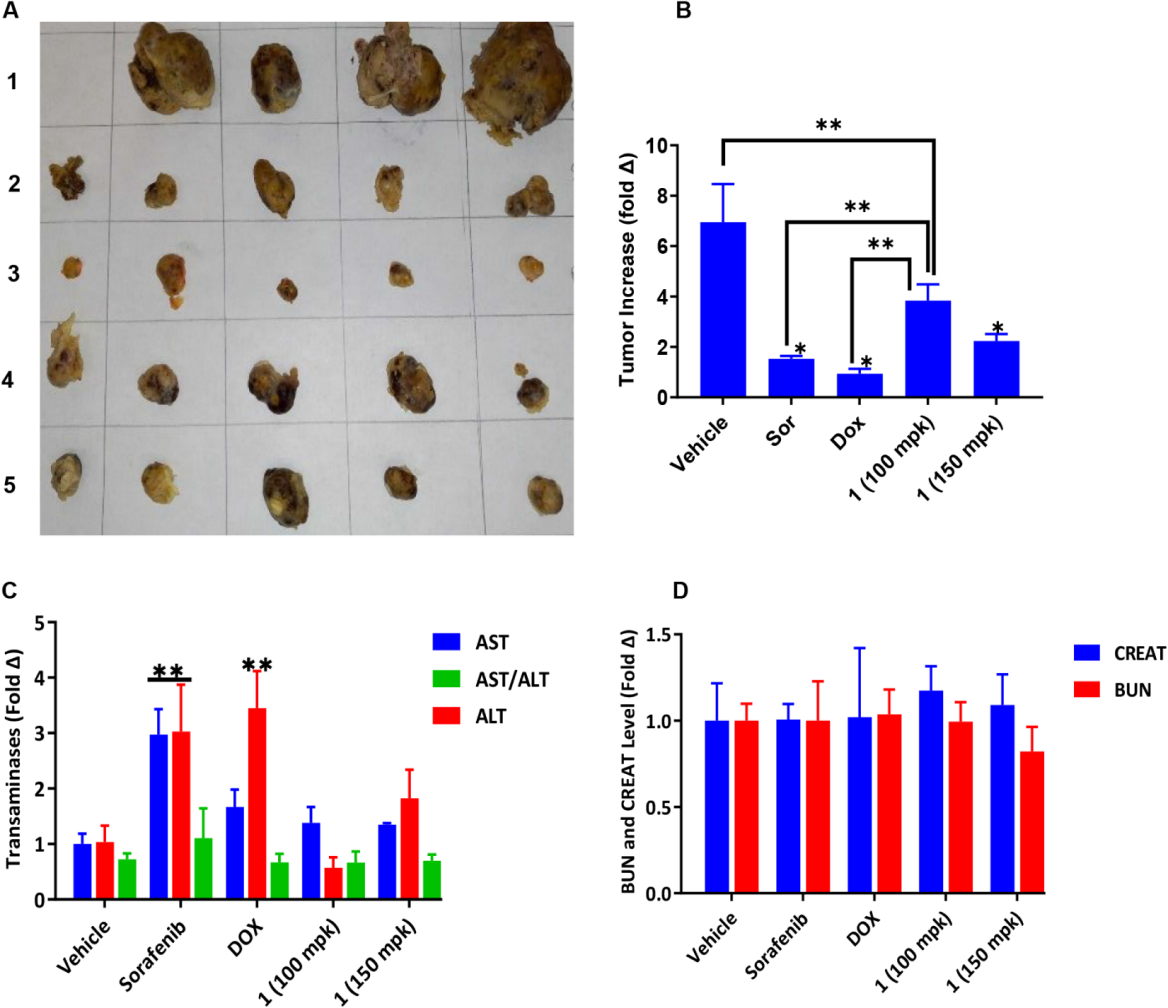
**A**. Antiproliferative activity of **1** on ectopic HCC xenografts in mice. (A, B) Images of excised tumors (A) and increase in tumor volumes (B) from mice treated with vehicle (1), Sorafenib (2/Sor), doxorubicin (3/Dox), and Compound **1** at 100 (4) and 150 (5) mpk. (C, D) Changes in the levels of AST, ALT, CREAT, and BUN in the plasma of mice treated with 1, Sor, and Dox relative to the average levels in vehicle-treated controls. Values represent mean ± standard deviation (*n* = 4 for vehicle and 5 for all other groups). Statistical significance was determined by ANOVA and Tukey-Kramer posthoc test: **p < 0.001, *p < 0.0001 (Vehicle vs treatment groups in panel B); **p < 0.001 (vehicle vs sorafenib/doxorubicin in panel C). ALT: alanine transaminase, AST: aspartate transaminase, BUN: blood urea nitrogen, and CREAT: creatinine.

### The Expression Levels of Genes Involved in Macroautophagy and Positive Regulation of Apoptosis Were Upregulated in HCC Cells Treated with Compound 1

Transcriptomics profiling of HCC cells treated with compound **1** was carried out using RNA sequencing (RNA-seq). The cells were treated in triplicate at 5 and 20 μM for 1, 6, and 24 h to enable concentration and time-based comparisons. A total of 33,285 genes with nonzero total read count (*p* < 0.05) across concentrations and time points were used for differential expression analysis, as shown in Figure S5 (Volcano and MA plots). The total number of annotated genes was 60,609. Close to 11 thousand genes (10,997) were found to be differentially expressed (Log_2_fold change (LFC), up and down) across time points (1, 6, and 24 h) when the DMSO-treated samples were compared with samples treated with 5 or 20 μM of **1.** The pairwise comparison revealed fewer differentially expressed genes (DEG, log2fold change) when cells were treated with 5 and 20 μM for 1 h compared to the 24-h treatment. The number of DEG at 1 h was 78 and 229, and at 24 h was 821 and 10,708 for 5 and 20 μM, respectively (Figure S6).

The total read counts per transcript were assessed and subsequently used to cross-compare transcriptomic profiles of the treated HepG2 cell lines within concentration and across time points. Multiple comparisons were made to verify that the top 30 transcriptomic profiles clustered in the same way, both in terms of dual-principal component variance (Figure S7) and heatmap (Figure S8), regardless of how groups were organized. The transcriptomic profiles of replicate samples from the same treatment cohort clustered closest to each other. At 1 h, the controls, 5 μM, and 20 μM treatment samples clustered together, but marked differences between the controls and treatment groups were observed at 6 and 24 h time points.

The top 30 differentially expressed genes observed across the various treatment time points are shown in Figure 9A–D and Table S1. The expressions of hmox1, abhd4, gabarapl1, akr1c1, and sqstm1 were significantly upregulated upon treatment with 5 and 20 μM of **1** for 6 h. Furthermore, the upregulation of CCN1 displayed a similar time-dependent increase when treated with 20 μM of **1**. Among the downregulated genes, DGKK, FREM1, ANGPTL8, and SLC13A5 were the most significantly affected. The top 30 differentially expressed genes across the time points were further analyzed using the DAVID gene system for functional annotation and enrichment analysis and through the Kyoto Encyclopedia of Genes and Genomes (KEGG) for functional clustering by gene ontology (GO) terms and pathway maps. The top 30 cellular pathways and molecular functions that match the dysregulated cellular genes are listed in Tables S2 and S3. These pathways were sorted by the number of candidate genes and matched to the pathways indicated. Since several identified pathways are functionally related, further assessment of pathway interconnectedness was necessary. This analysis was performed using the DICE-Tools GO-database, GOnet. Various biological processes, including macroautophagy, PRAP, cell migration and motility, secreted and extracellular proteins (Figure 10), as well as over 150 GOnet interconnected (Figures S9 and S10) pathways, were identified using the top 30 genes (Tables S2, S3, and Figure S6). The enriched pathways identified were associated with positive regulation of cell migration for biological processes, but none were identified for both cellular processes and molecular function (Figure S9)

**Figure 9:**
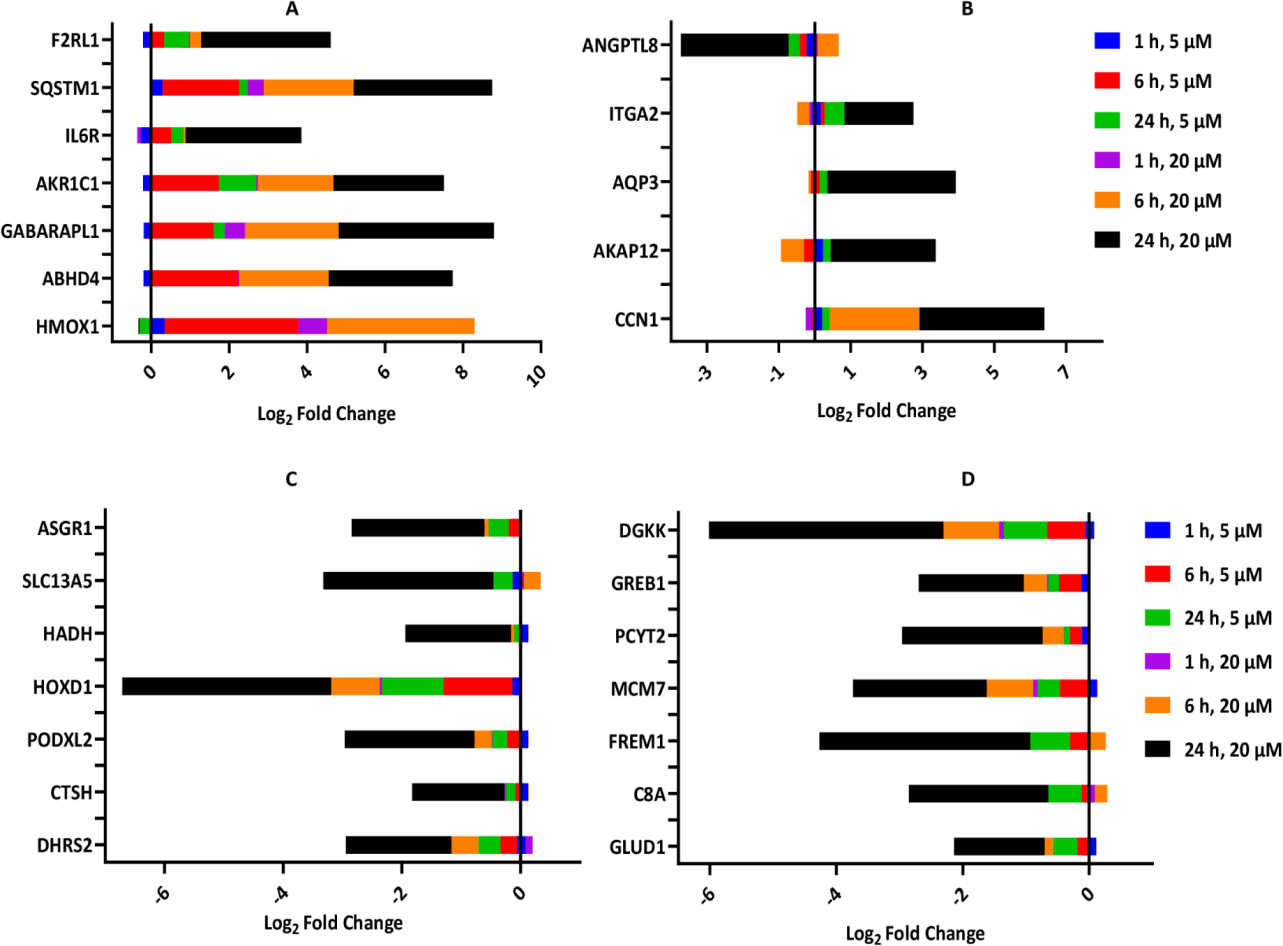
Transcriptomics analysis of Hep G2 cells treated with Compound **1**. The graphs show the top upregulated (A, B) and downregulated genes (C, D) at 1, 6, and 24 h in cells treated with 5 and 20 μM of **1** relative to DMSO-treated control cells. *n* = 3 in all.

**Figure 10:**
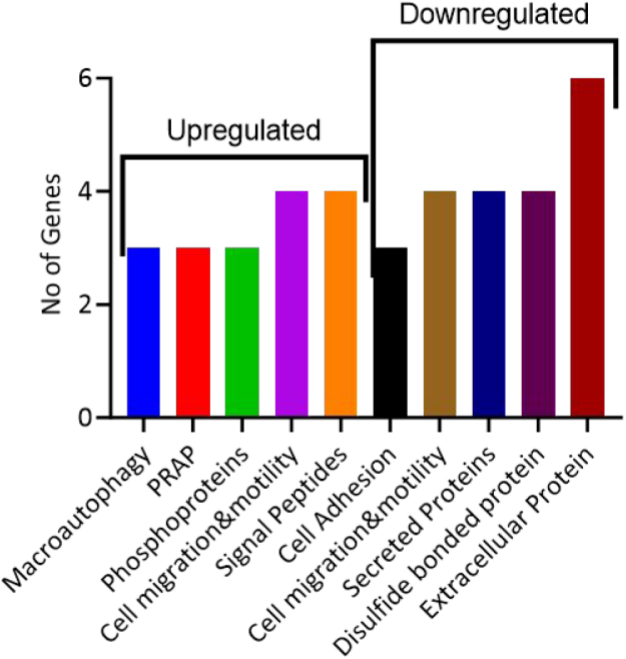
Selected biological processes of differentially expressed genes when Hep G2 cells were treated with 20 μM of 1 for 24 h. PRAP: positive regulation of apoptosis.

Follow-up qRT-PCR validation assays show that the levels of gabarapl1, akr1c1, and sqstm1 were significantly increased at 5 and 20 μM for 6 h, and at 20 μM for 24 h (Figure 11). The expression level of akap12 was also significantly upregulated at both concentrations at 24 h. There was no notable change in angptl8 levels after 6 h at either concentration, but qRT-PCR data revealed a modest decrease in angptl8 levels at 20 μM after 24 h of treatment. Western blot analysis to investigate whether treatment with **1** induced apoptosis revealed a marked increase in cleaved PARP1 levels, which was concentration- and time-dependent. Similarly, cleaved caspase 3 levels were elevated 24 h after treatment with 5 and 20 μM of compound **1** (Figure 12).

**Figure 11:**
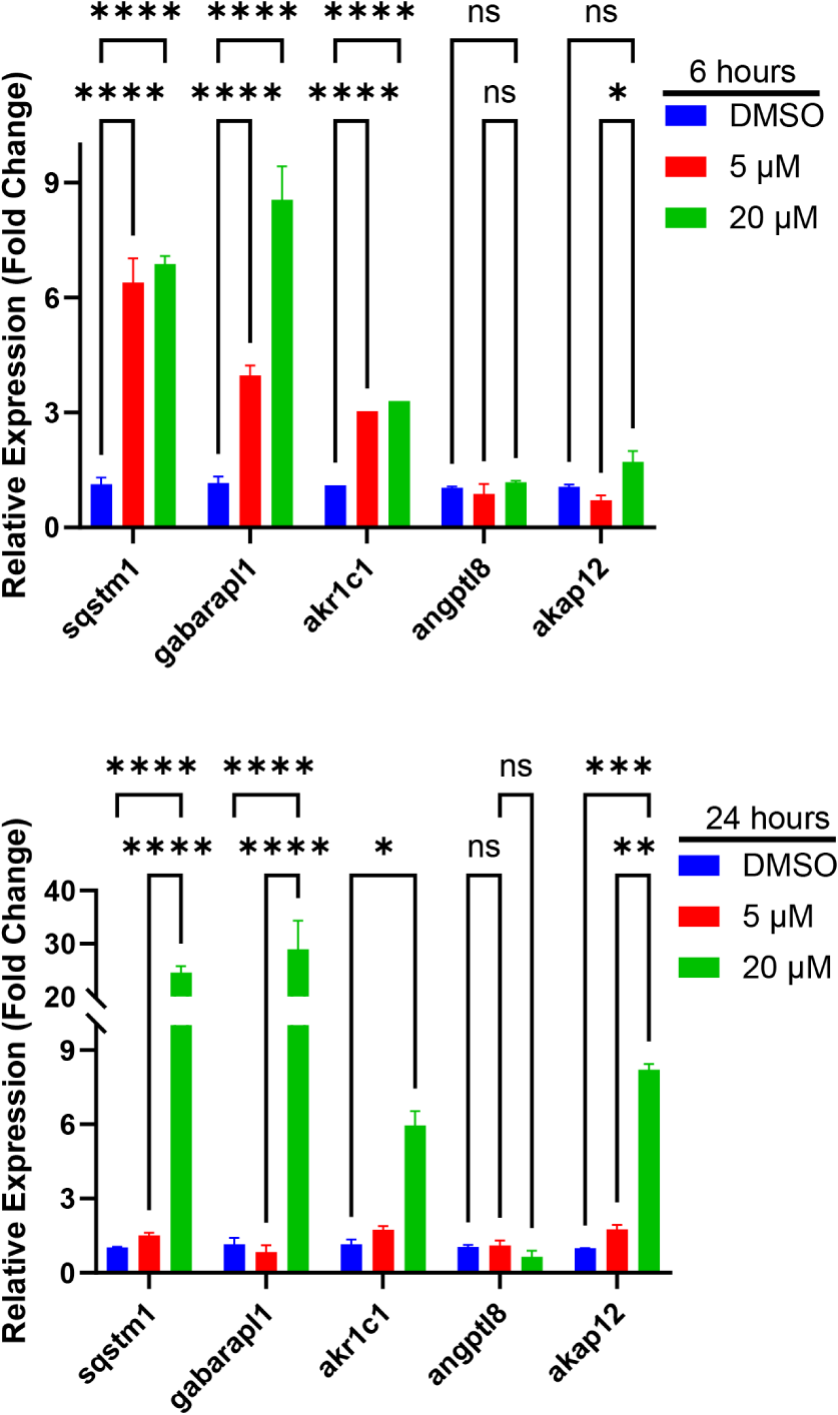
Effect of Compound **1** on gene expression in Hep G2 cells. RT-PCR analysis of sqstm1, gabarapl1, akr1c1, angptl8, and akap12 in cells treated for 6 (top) or 24 (bottom) hours. *N* = 2. Data are represented as mean ± SD of two biological replicates. Statistical significance was determined by ANOVA and Tukey-Kramer posthoc test: ****p < 0.0001, **p < 0.001; *p < 0.05; ns = not significant.

**Figure 12:**
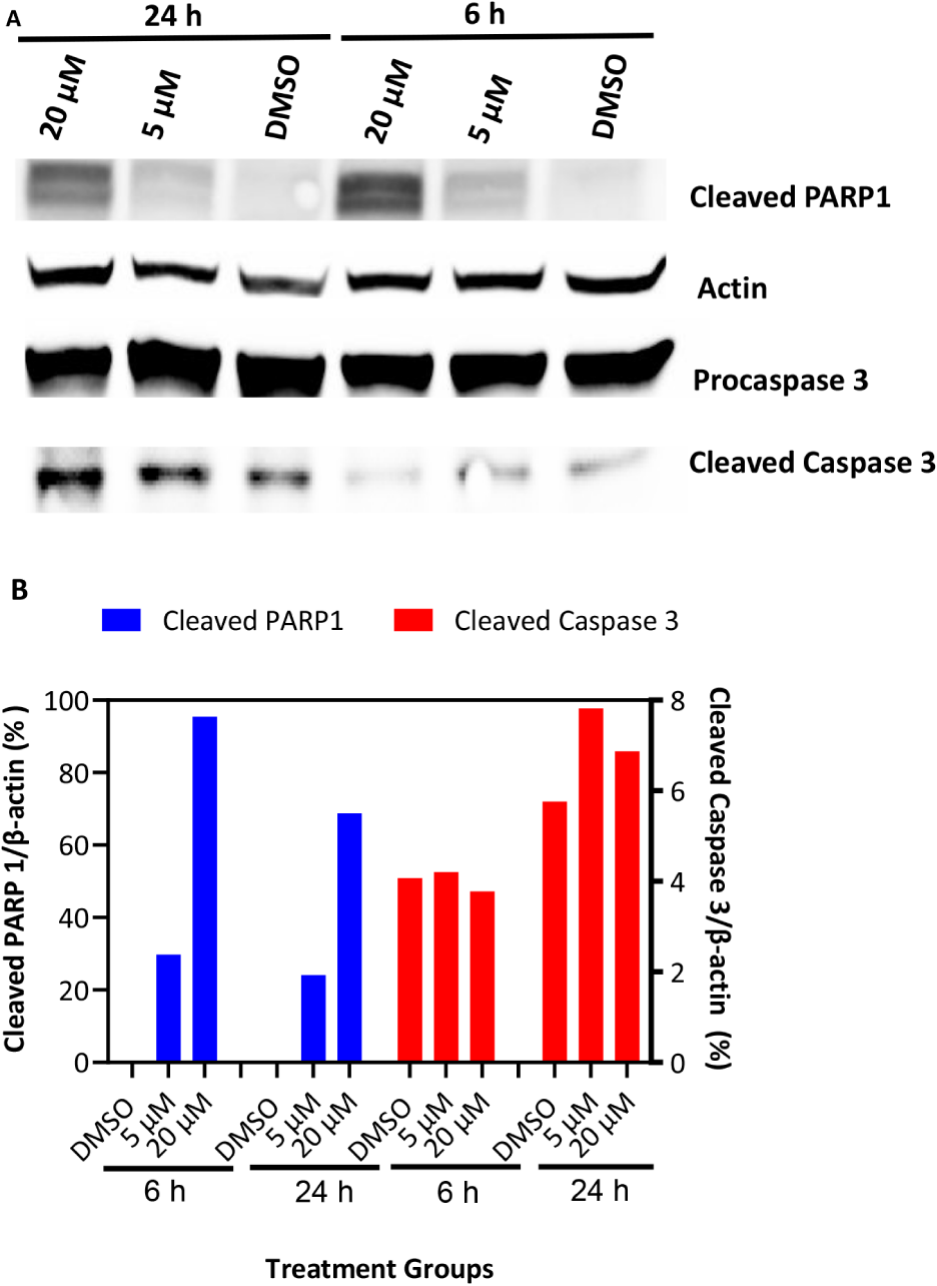
Compound **1** activates apoptosis in Hep G2 cells. (A, B) Representative Western blot of cleaved PARP1 and Caspase 3 in Hep G2 cell lysates and quantitative analysis of band intensity. The cells were treated for 6 or 24 h, and extracted lysates were probed with a cocktail of primary antibodies for cleaved PARP1, procaspase 3, cleaved Caspase 3, and actin.

## Discussion

Inhibitors of cathepsins have been investigated for a variety of diseases, including cancers, but none of the reported inhibitors have shown significant antiproliferative potency against hepatocellular carcinoma.^16,28–32^ Since upregulation of cathepsin L is linked to aggressive proliferation of HCC *in vitro*, and CatS has been shown to be aberrantly overexpressed in HCC while the silencing of CatS induces apoptosis in HCC, inhibiting both proteases could affect the proliferation of HCC cells.^14,15,33–35^ Based on the results outlined above, the antiproliferative activity of compounds **1** and **2** against HCC is, at least, in part due to the inhibition of CatL and CatS. Since most reported CatL and CatS inhibitors lack significant antiproliferative activity despite their potency on the proteases, and the inhibitory activities of both compounds on recombinant cathepsin L and S were significantly higher than the activity on cathepsins in the cell lysates, we envisage that there is likely an additional mode of action(s) for compounds **1** and **2.** ^17,18,31,36^ Treatment of Hep-G2 cells with potent cathepsin inhibitor, E-64d, which effectively inhibit cathepsin S and L activity *in vitro*, does not produce comparable antiproliferative effects (Figure S11). Consequently, the potential of reactive oxygen species (ROS) generation due to the presence of the nitroaromatic moiety, a metabolic liability, in the compounds was investigated. However, the results showed that the levels of ROS generated in cells treated with compounds **1** and **2** were not significantly different from those of DMSO-treated controls over a 24-h period, and the levels were significantly less than those of the positive control (H_2_O_2_).^37–39^ Additional target validation and structure–activity relationship studies to address the metabolic liabilities are underway, and the results will be reported in due course. The effect of compound **1** on subcutaneous xenograft tumors in nude mice showed marked reductions in tumor mass compared to controls, but the reduction observed in compound **1**-treated animals was inferior to the effect observed with Sorafenib and doxorubicin. However, the animals treated with compound **1** were less lethargic and had significantly lower levels of plasma ALT compared to the Sorafenib and doxorubicin groups. Despite significant advances in HCC chemotherapy, systemic toxicity of standard-of-care therapies remains a very persistent issue. When compared with doxorubicin and Sorafenib, treatment with **1** resulted in prolonged survival and moderate weight gain in mice, demonstrating its tolerability (Figure S4). Profiling of the transcriptome in HCC cells treated with **1** revealed significant changes in the expression of genes involved in the regulation of cell death, cell proliferation, and locomotion, which could account for the antitumor effects of compound **1**. Some of the differentially expressed genes in this work have been investigated in the context of the proliferation, tumorigenesis, drug resistance, and metastasis of HCC. The upregulation of sqstm1, gabarapl1, and akr1c1 across time points and concentrations strongly suggests an increase in autophagic flux in treated cells.^40–42^ Elevated cleaved PARP1 and cleaved caspase 3 indicate enhanced apoptotic signaling. Gao and coworkers have shown that upregulation of *angptl8* correlates with increased HCC malignancy, poor prognosis, and enhanced tumor cell proliferation and immune escape.^43^ The transcriptome shows a significant downregulation of *angptl8* expression in cells treated with **1** at 20 μM, which correlates with its antiproliferative activity. Similarly, *slc13a5* plays a significant role in energy homeostasis in HCC, and silencing of *slc13a5* has been shown to suppress the proliferation of Hep G2 cells and tumor growth in mice.^44,45^ Also, MCM7 expression was significantly downregulated in the Hep G2 cells treated with **1.** MCM7, a part of the MCM2–7 helicase complex that is essential for initiating DNA replication, is associated with increased tumorigenicity and has been shown to contribute to poor prognosis in HCC patients.^46^ Overall, the cathepsin inhibitor (**1**) displayed promising *in vitro* and *in vivo* antiproliferative activities. It is well tolerated at the administered doses (100 and 150 mg/kg) in mice, but additional structure optimization is needed to improve bioavailability and potency.

## Supplementary Material



## Notes

Additional experimental details and methods, including **Scheme S1**, **Figures S1–S11**, and **Table S1–S3** (PDF)

## Abbreviations

HMOX1: heme oxygenase-1
ABHD4: abhydrolase domain containing 4, *N*-acyl phospholipase B
GABARAPL1: GABA type A receptor-associated protein-like 1
AKR1C1: aldo-keto reductase family 1 member C1
SQSTM1: sequestosome 1
CCN1: cellular communication network factor 1
DGKK: diacylglycerol kinase kappa
FREM1: FRAS1 (faser extracellular matrix complex subunit 1)
ANGPTL8: angiopoietin-like 8
AKAP12: A-kinase anchoring protein 12
SLC13A5: solute carrier family 13 member 5
HOXD-AS1: homeobox D1
C8A: complement C8 alpha chain
IL6R: interlukin 6 receptor
MCM7: minichromosome maintenance complex component 7
