# Supplementary material for: Cathepsin Inhibitor Suppresses the Growth of Ectopic Hepatocellular Carcinoma Tumors in Mouse Models
Source: ACS Pharmacol Transl Sci. 2026 Mar 16;9(4):934–46. doi: 10.1021/acsptsci.5c00675 (PMC13077493; doi:10.1021/acsptsci.5c00675)
Supplement: Supplementary file 1 [file pt5c00675_si_001.pdf]

Supporting Information

Cathepsin Inhibitor Suppresses the Growth of Ectopic Hepatocellular Carcinoma Tumors in Mouse Models

Olamide Crown,[1] Oluwatoyin V. Odubanjo,[2] Olawale S. Adeyinka,[1] Felicite K. Noubissi,[2] Ifedayo Victor Ogungbe[1*]

[1]Chemistry and Biotechnology Science and Engineering Programs, The University of Alabama in Huntsville, 301 Sparkman Drive, Huntsville, Alabama 35899, USA

[2]Department of Biology, Jackson State University, Jackson, Mississippi 35217, USA

***Corresponding Author: Victor.Ogungbe@uah.edu**

Table of contents

Content	Page Number
Synthesis and Characterization of Compounds 1 and 2	S2-4
Inhibition Plots of cathepsin L and S by 1 and 2	S5
Images of Hep G2 cells treated with 1 (1-50 µM) or DMSO (Vehicle) for 24 hours after pretreatment with 50 µM H2DCFDA	S6 and S7
Xenograft tumor volume and body weight of mice treated with Compound 1 and survival curves	S8
The Volcano and MA plots of the differentially expressed genes in Hep G2 cells treated with 1 across time points	S9
The dispersion plot of differentially expressed genes	S10
The dual-principal component variance plots generated from the transcriptomics analysis of Hep G2 cells treated with compound 1	S11
Heatmap showing the hierarchically clustered Euclidean distances between samples, based on the regularized log transformation of the normalized count data.	S12
List of the top thirty differentially expressed genes across the time points	S13
List of the biological processes of differentially expressed genes when Hep G2 cells were treated with 1.	S14 and S15
List of the molecular functions of differentially expressed genes when Hep G2 cells were treated with 1.	S16 and S17
The enriched pathway linked to the differentially expressed genes in the cells treated with 1 across the time points.	S18
The interconnected pathway linked to the differentially expressed genes in cells treated with 1 across time points.	S19
Plot of the antiproliferative activity of E-64d on Hep G2 cells	S20

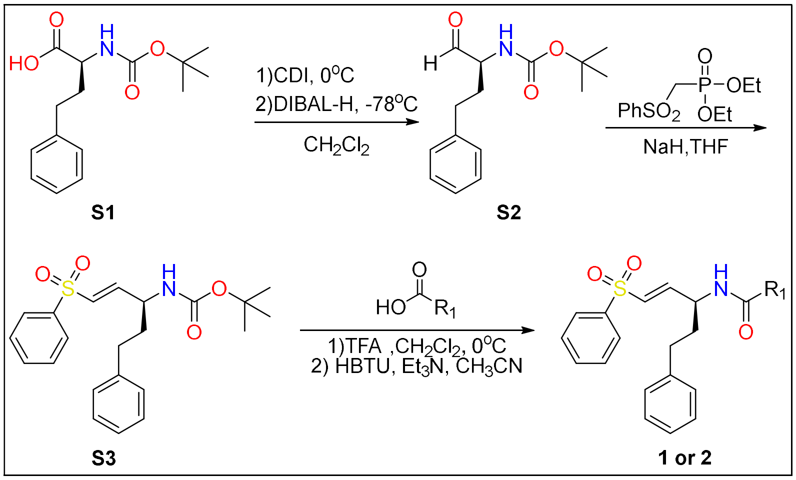

Scheme S1. Synthesis of target compounds **1 and 2**

Synthesis of S1

A dichloromethane solution (20 mL) of Boc-homophe-OH (1.397 g, 50 mmol) was treated with 1,1'-carbonyldiimidazole (recrystallized in tetrahydrofuran) at 0°C for 60 min. Subsequently, DIBAL-H (10.5 mL, 105 mmol) was added dropwise at -78°C.[10] TLC analysis showed full consumption of the starting material. The reaction mixture was quenched with 10% tartaric acid and diluted with ethyl acetate and saturated with sodium chloride. The layers were separated, followed by the extraction with aqueous ethyl acetate. The organic layer was dried over $MgSO_4$, filtered, and evaporated. The mixture was purified by flash column chromatography to obtain compound **S1** (55 % yield).

Synthesis of S2

Sodium hydride (1.5 eq) in tetrahydrofuran was cooled to 0°C, and diethyl((phenylsulfonyl)methyl) phosphonate in tetrahydrofuran was added dropwise. The reaction was stirred at 0̊C for 20 min, then compounds **S2** in tetrahydrofuran were added. The reaction was quenched with a saturated solution of sodium bicarbonate after 5 minutes of stirring. The mixture was diluted with ethyl acetate and water. The layers were separated, followed by the extraction of the aqueous phase with ethyl acetate. The organic layer was dried over $MgSO_4$, filtered, and evaporated. The mixture was purified by flash column chromatography to obtain compound **S3** (70 % yield).

Synthesis of 1 and 2

Compound **S3** (0.1 mmol) was dissolved in a 33% TFA: dichloromethane solution (1.5 mL) and allowed to stir at a 0˚C for 1.5 h. The solution was concentrated and washed twice with 5 mL of toluene to remove

the remaining TFA. The deprotected amine was then dissolved in 2 mL of anhydrous acetonitrile, and 5-nitro-2-furoic acid or 5-nitrothiophene-2-carboxylic acid (0.10 mmol), triethylamine (Et_3N, 31 µL, 0.218 mmol), and hexafluorophosphate benzotriazole tetramethyl uronium (HBTU, 41.7 mg, 0.11 mmol) were added. The solution was allowed to stir overnight. The reaction was quenched with brine (5 mL). The aqueous layer was extracted three times with 10 mL of ethyl acetate. The extract was concentrated and meticulously purified on prep-TLC plates. The products were characterized using NMR and high-resolution mass spectrometry. All reagents were purchased from commercial sources and used without further purification. The ^{1}H and ^{13}C NMR spectra were recorded on a Bruker Ultrashield Avance 400 (Bruker, Billerica, MA, USA) spectrometer. Thin-layer chromatography (TLC) and NMR were used to monitor reactions and purification conditions. Compounds were meticulously purified by column chromatography on silica gel or on pre-coated preparative TLC plates. Accurate mass information was obtained using a Synapt G2 HDMS instrument operated in either positive or negative ESI mode. HPLC-UV analysis was carried out on a Shimadzu Prominence HPLC-UV system using isocratic elution (Pinnacle II C18 5 µM, 200 x 4.6 mm column; flow rate = 0.5 mL/min) with 65% MeOH and 35% H_2O for **1** and 90% MeOH, 5% ACN, and 5% H_2O for **2**.

(*S*,*E*)-5-nitro-*N*-(5-phenyl-1-(phenylsulfonyl)pent-1-en-3-yl)furan-2-carboxamide (1)

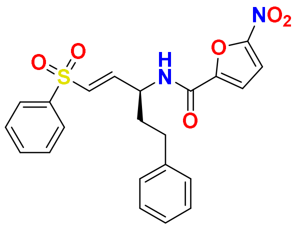

^{1}H NMR (500 MHz, $CDCl_3$) δ 7.86 (d, J = 7.5 Hz, 2H), 7.63 (t, J = 7.5 Hz, 1H), 7.54 (t, J = 7.5 Hz, 2H), 7.35 (d, J = 3.5 Hz, 1H), 7.23 (d, J = 8.0 Hz, 1H), 7.22 (d, J = 3.5 Hz, 1H), 7.16 (t, J = 7.5 Hz, 1H), 7.12 (d, J = 7.5 Hz, 2H), 6.97 (dd, J = 15.0 Hz, J = 5.0 Hz, 1H), 6.66 (d, J = 8.5 Hz, 1H), 6.47 (dd, J = 15.0 Hz, J = 1.5 Hz, 1H), 4.88 (m, 1H), 2.73 (t, J = 7.5 Hz, 2H), 2.16-2.06 (m, 2H). ^{13}C NMR (125 MHz, $CDCl_3$) δ 156.0, 151.3, 147.3, 144.5, 140.1, 139.7, 133.8, 131.4, 129.5, 128.8, 128.4, 127.8, 126.4, 116.6, 112.5, 49.6, 35.2, 32.2. HRMS $[M-H]^+$ calculated for $C_{22}H_{21}N_2O_6S$: 441.1120; found: 441.1126. HPLC-UV: RT = 13.55 min (97.9%).

(*S*,*E*)-5-nitro-*N*-(5-phenyl-1-(phenylsulfonyl)pent-1-en-3-yl)thiophene-2-carboxamide (2)

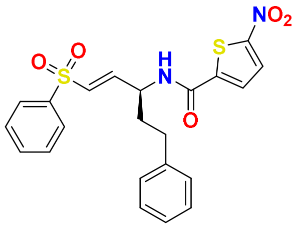

^{1}H NMR (500 MHz, $CDCl_3$) δ 7.86 (d, J = 9.0 Hz, 2H), 7.75 (d, J = 5.5 Hz, 1H), 7.66 (tt, J = 9.0 Hz, J = 1.5 Hz, 1H), 7.56 (t, J = 9.5 Hz, 2H), 7.28 (t, J = 8.5 Hz, 2H), 7.21 (t, J = 9.5 Hz, 1H), 7.14 (d, J = 9.0 Hz, 2H), 7.11 (d, J = 5.0 Hz, 1H), 7.00 (dd, J = 19.0 Hz, J = 6.5 Hz, 1H), 6.47 (dd, J = 19.0 Hz, J = 2.0 Hz, 1H), 6.38 (d, J = 11.0 Hz, 1H), 4.93 (m, 1H), 2.75 (td, J = 9.0 Hz, J = 2.5 Hz, 2H), 2.12 (t, J = 9.0 Hz, 2H). ^{13}C NMR (125 MHz, Acetone-d_6) δ 160.2, 154.4, 146.1, 146.0, 141.5, 141.4, 134.0, 131.6, 129.9, 129.6, 128.92, 128.91, 128.0, 127.6, 126.5, 50.7, 35.6, 32.5. HRMS $[M\text{-}H]^+$ calculated for $C_{22}H_{21}N_2O_5S_2$: 457.0892; found: 457.0892. HPLC-UV: RT = 4.73 min (99.9%).

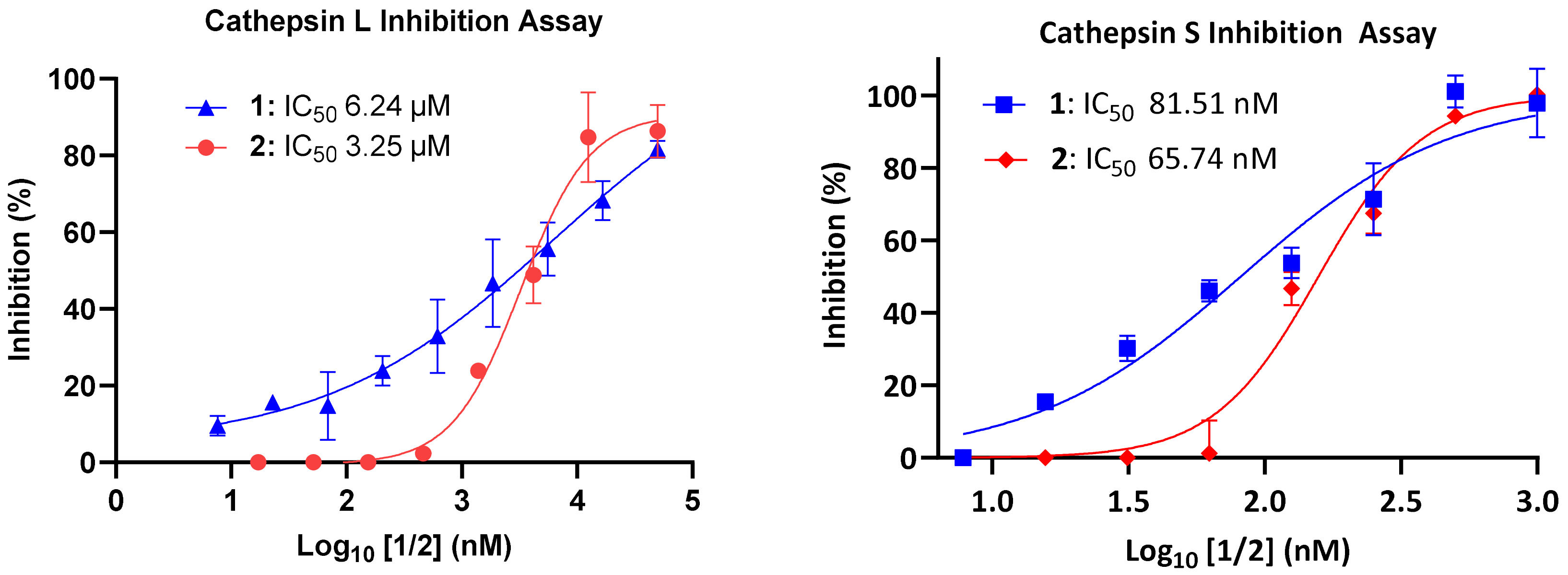

Figure S1: Inhibition of cathepsin L (right) and S (Left) by 1 and 2. The enzymes and inhibitors were preincubated for an hour before the substrates (Z-FR-AMC for CatL and Z-VVR-AMC for CatS) were added. The substrates' hydrolysis was monitored at $\lambda_{ex}/\lambda_{em}$ 355/460 nm on a PolarStar Omega plate reader (BMG LABTECH, Germany) as described in the methods section. n = 3 for all concentrations.

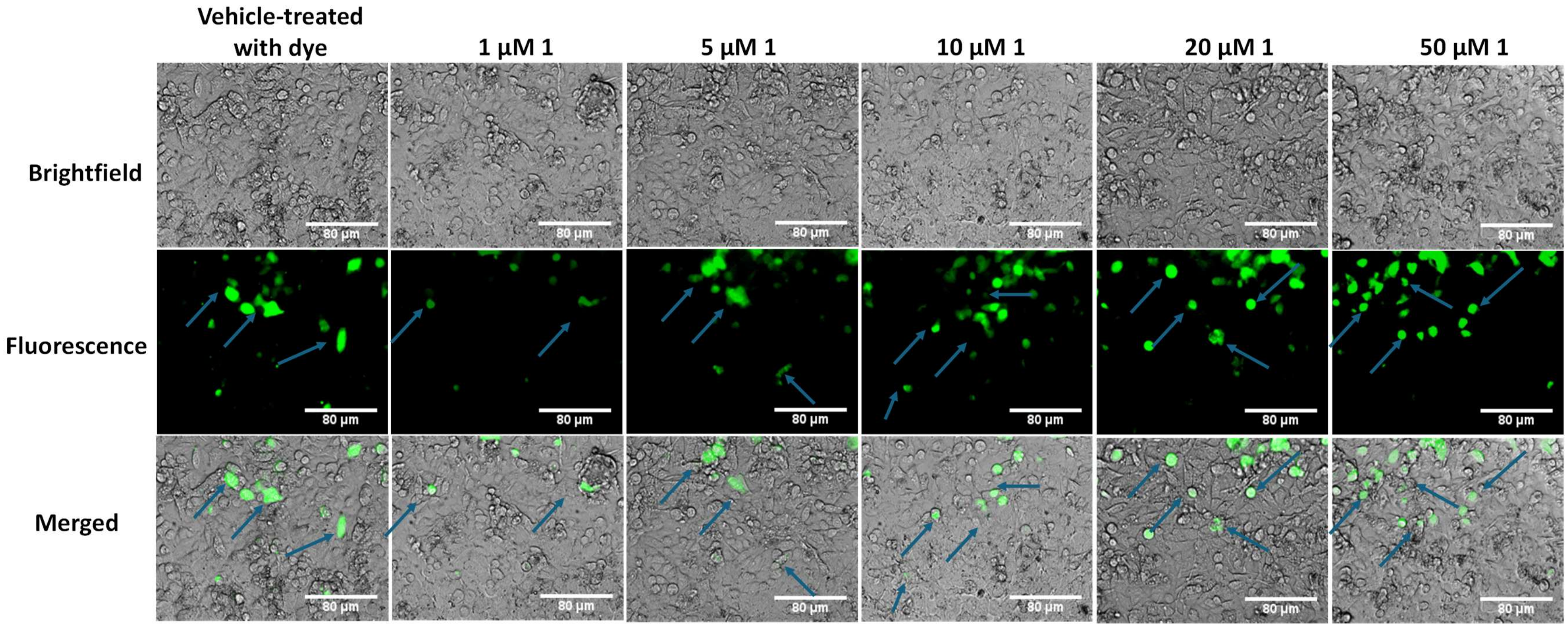

Figure S2: Images of Hep G2 cells (50,000 cells/well) treated with different concentrations of 1 (1-50 µM) or DMSO (Vehicle) for 24 hours. The cells were pretreated with 50 µM H2DCFDA for 30 minutes before compound **1** was added. The blue arrows point to the green fluorescence emitted by H2DCFDA.

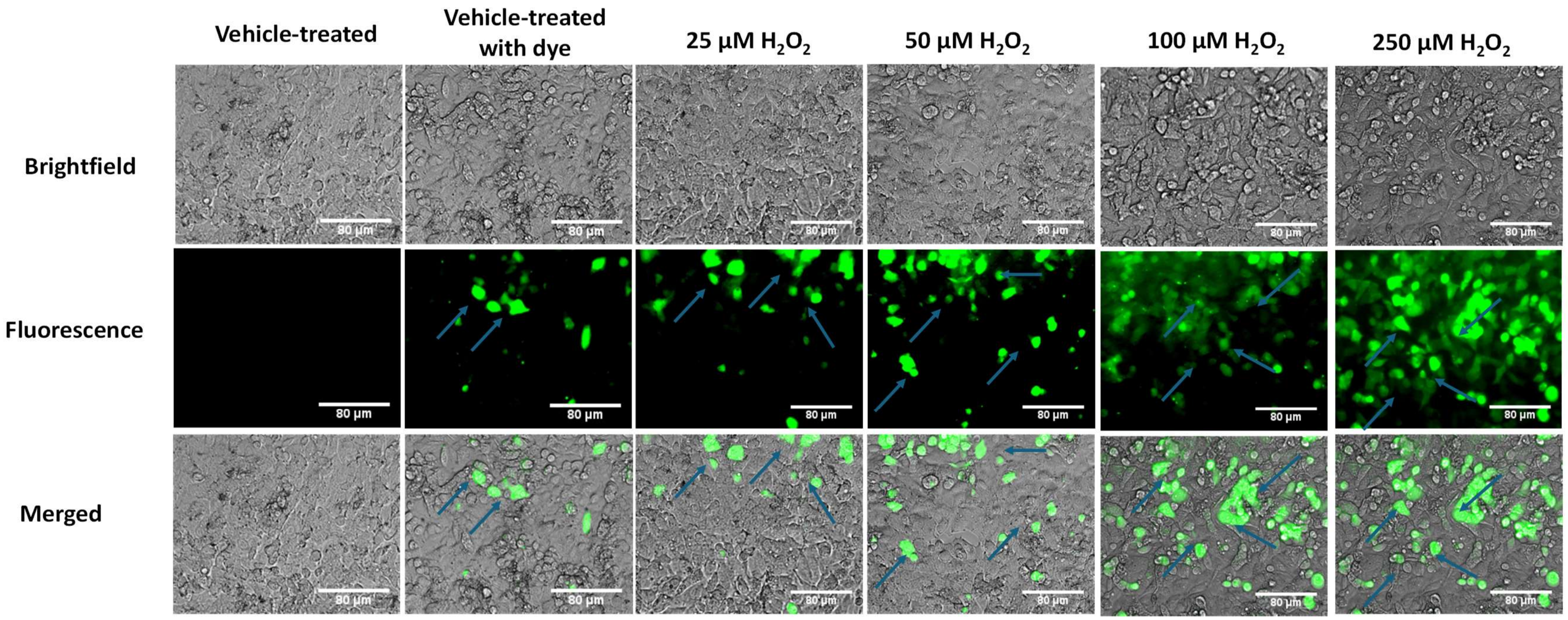

Figure S3: Images of Hep G2 cells (50,000 cells/well) treated with different concentrations of H_2O_2 (25-200 µM) or DMSO (Vehicle) for 24 hours. The cells were pretreated with 50 µM H2DCFDA for 30 minutes before compound **1** was added. The blue arrows point to the green fluorescence emitted by H2DCFDA.

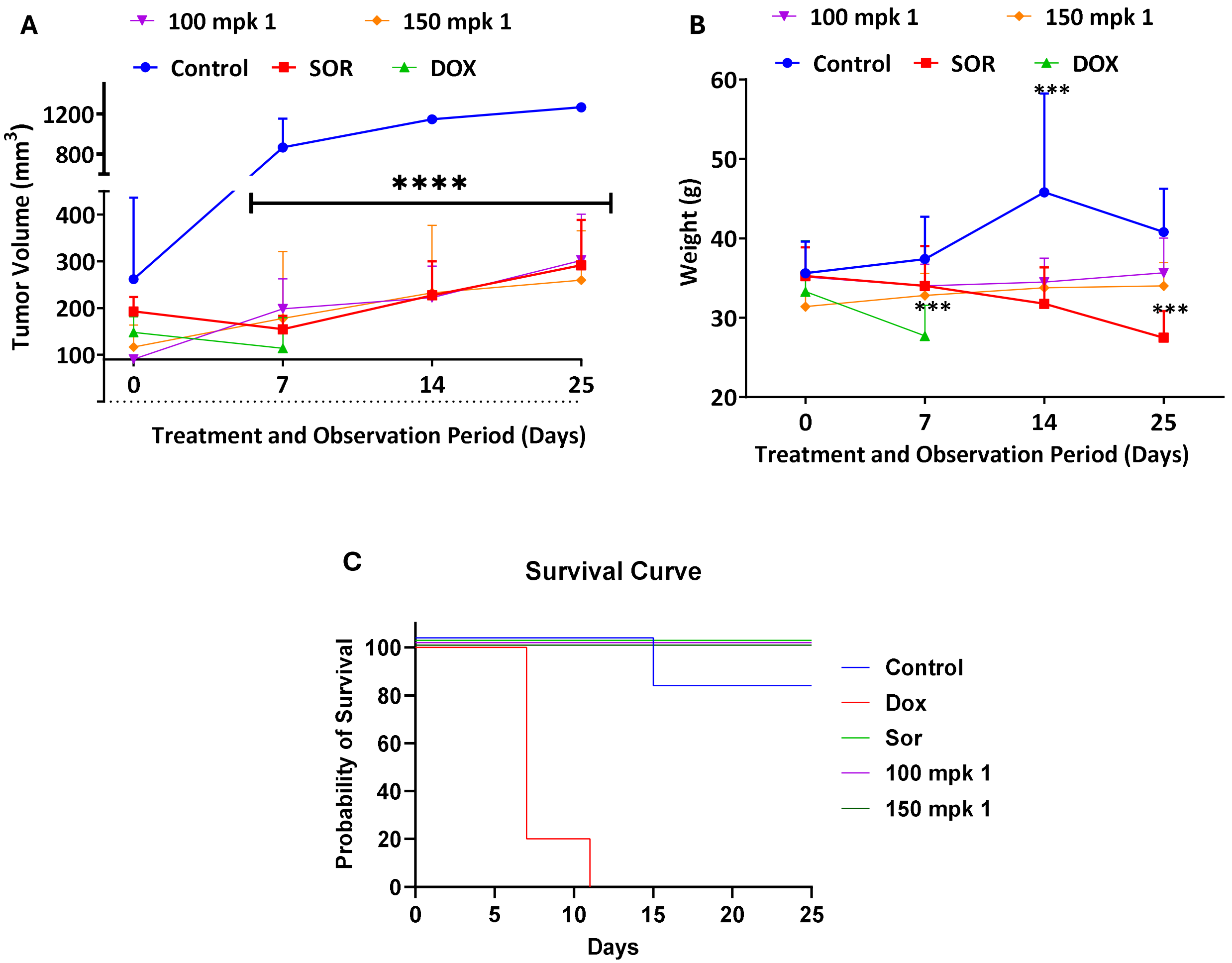

Figure S4: Changes in xenograft tumor volume (A) and body weight (B) of mice treated with Compound 1 and the survival curves (C). The results **A** and **B** are presented as mean ± SD (n = 5) and analyzed with ANOVA and Tukey-Kramer post-hoc test. . **** and *** indicate statistically significant differences at $p < 0.0001$ and $p < 0.001$, respectively, between 100 and 150 mpk of 1, SOR (30 mpk) and DOX (7.5 mpk) compared to vehicle-treated controls. SOR: Sorafenib; DOX: Doxorubicin.

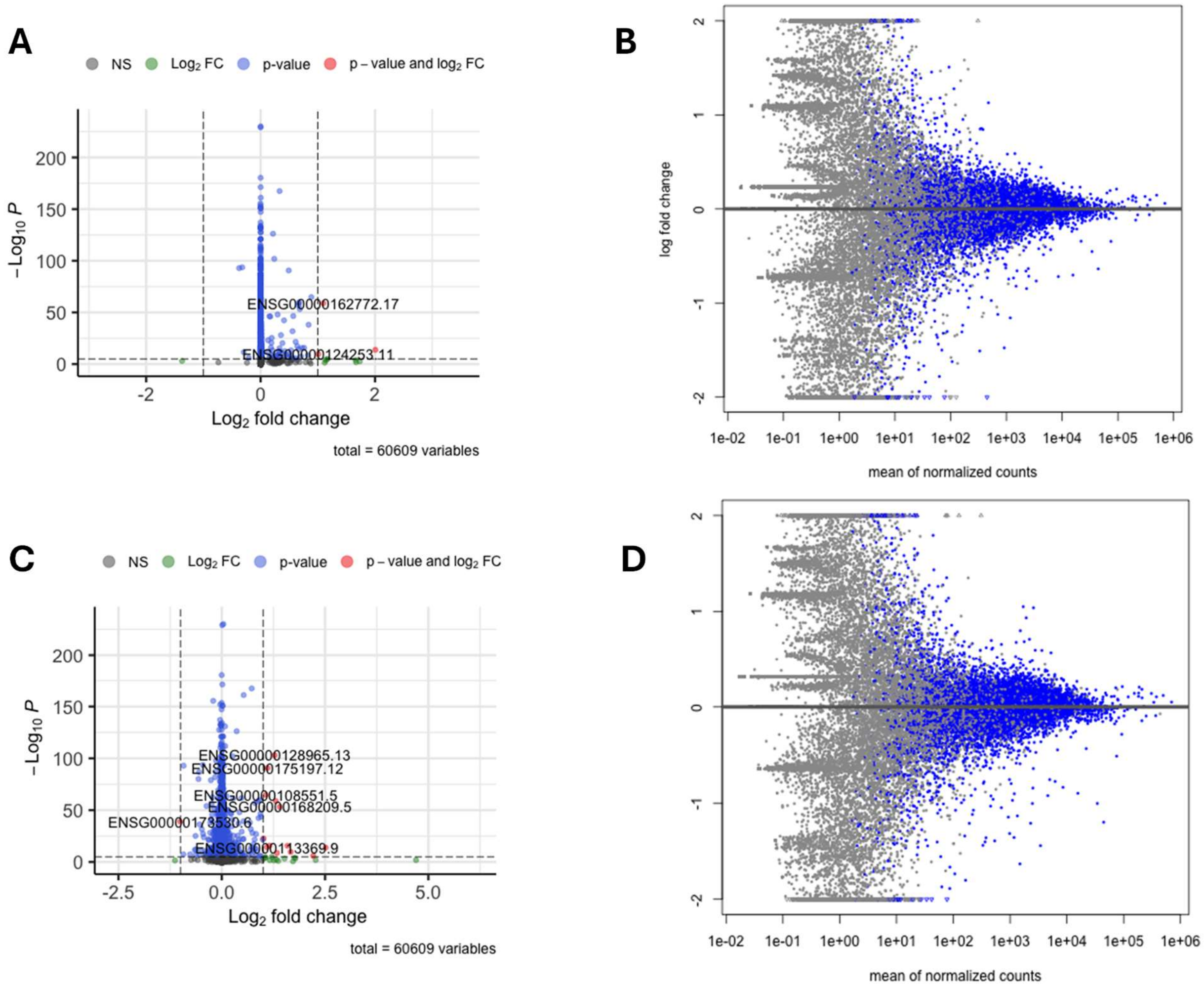

Figure S5: The Volcano and MA plots of the differentially expressed genes in Hep G2 cells treated with 1 across time points. **A and B**. The Volcano and MA plots of cells treated with 5 µM of 1 across timepoints. **C and D.** The Volcano and MA plot of cells treated with 20 µM of 1 across timepoints. The MA plot displays the relationship between the magnitude of differential expression and the average expression across all samples for each gene.

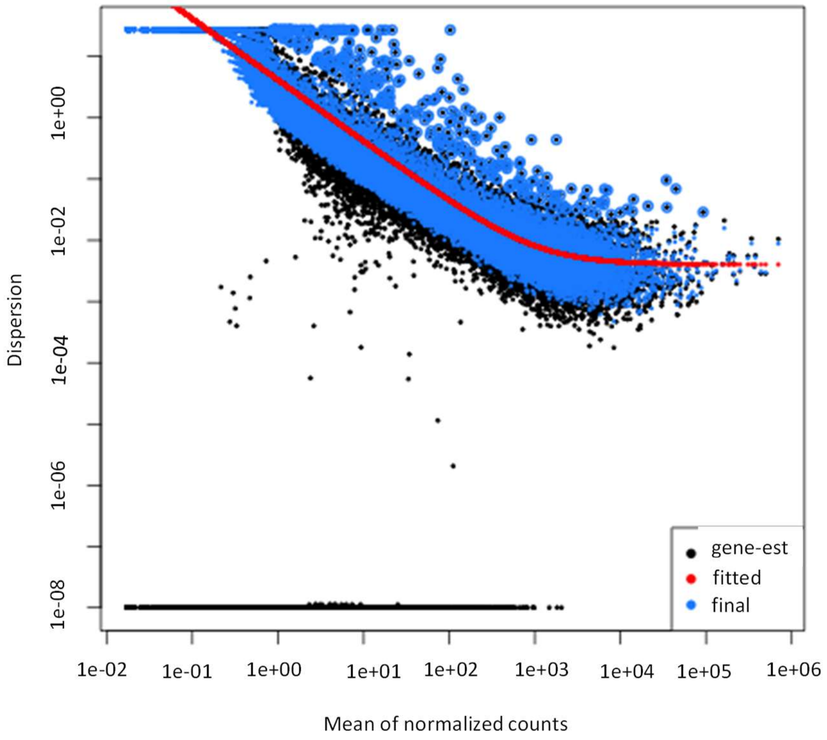

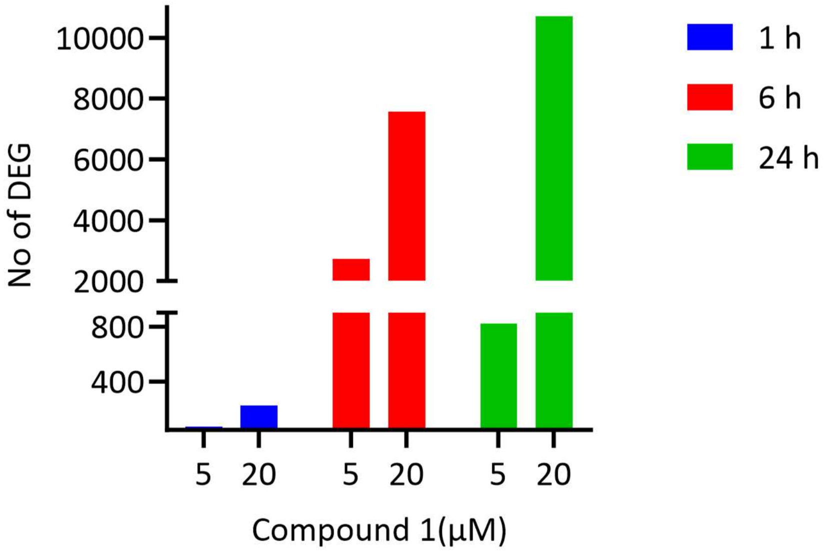

Figure S6: The dispersion plot shows the shrinkage of the differentially expressed genes (blue dots) towards the curve Left). While on the left is the total amount of differentially expressed genes per each time point. Key: DEG-Differentially Expressed Genes.

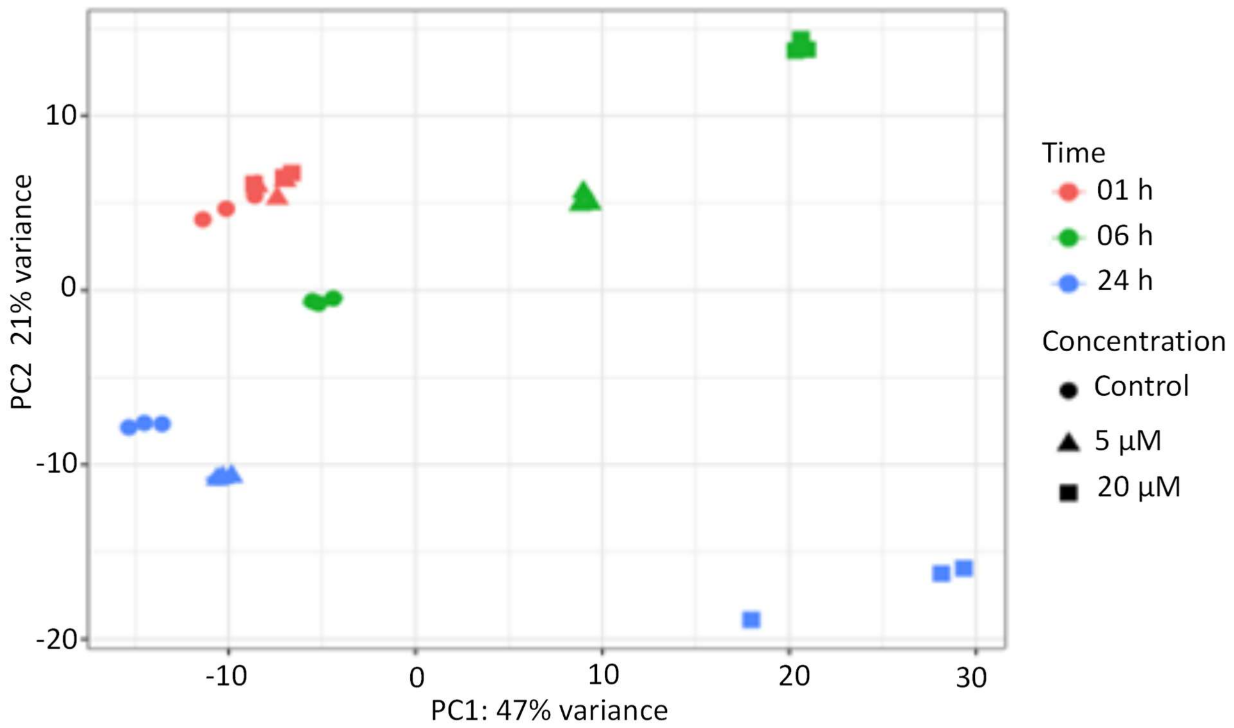

Figure S7: The dual-principal component variance plots generated from the transcriptomics analysis of Hep G2 cells treated with compound 1 at 5 and 20 µM for 1, 6, or 20 hours.

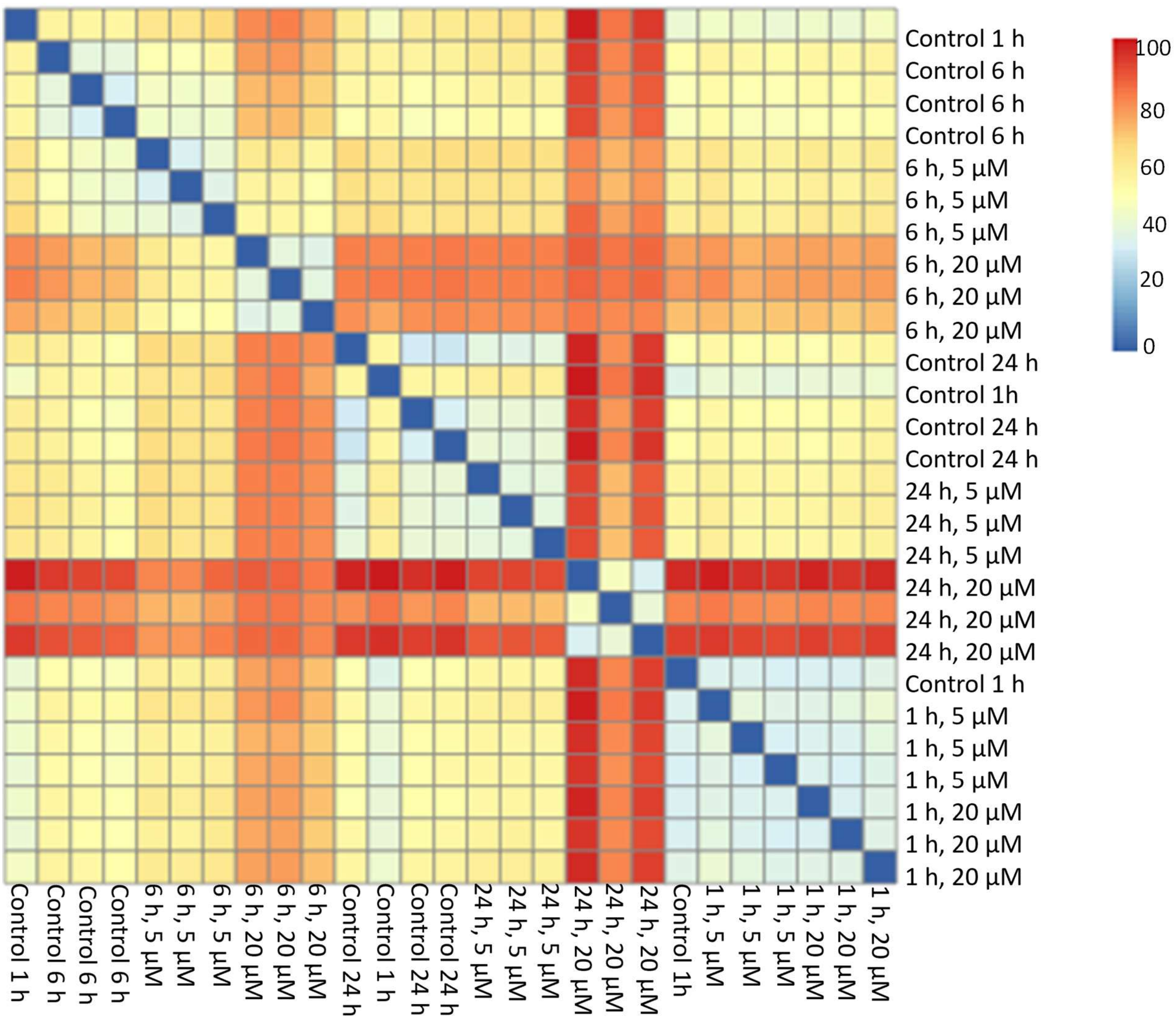

Figure S8: Heatmap showing the hierarchically clustered Euclidean distances between samples from the regularized log transformation of the normalized count data.

Table S1: List of top thirty differentially expressed genes across the time points (1, 6, and 24 hours) and concentrations (5 and 20 μM) of **1**

Gene ID	Gene	Gene name
**ENSG00000051108.15**	HERPUD1	Homocysteine inducible ER protein with ubiquitin like domain 1
**ENSG00000100292.17**	HMOX1	Heme oxygenase 1
**ENSG00000100439.10**	ABHD4	Abhydrolase domain containing 4, N-acyl phospholipase B
**ENSG00000100867.14**	DHRS2	Dehydrogenase/reductase 2
**ENSG00000103811.16**	CTSH	Cathepsin H
**ENSG00000108819.11**	PPP1R9B	Protein phosphatase 1 regulatory subunit 9B
**ENSG00000114631.11**	PODXL2	Podocalyxin like 2
**ENSG00000120053.12**	GOT1	Glutamic-oxaloacetic transaminase 1
**ENSG00000128645.15**	HOXD1	Homeobox D1
**ENSG00000130173.13**	ANGPTL8	Angiopoietin like 8
**ENSG00000131016.17**	AKAP12	A-kinase anchoring protein 12
**ENSG00000138796.16**	HADH	Hydroxyacyl-CoA dehydrogenase
**ENSG00000139112.11**	GABARAPL1	GABA type A receptor associated protein like 1
**ENSG00000141485.16**	SLC13A5	Solute carrier family 13 member 5
**ENSG00000141505.12**	ASGR1	Asialoglycoprotein receptor 1
**ENSG00000142871.17**	CCN1	Cellular Communication Network Factor 1
**ENSG00000143013.13**	LMO4	LIM (Lin-11, Islet-1, and Mec-3) Domain only 4
**ENSG00000148672.9**	GLUD1	Glutamate Dehydrogenase 1
**ENSG00000157131.10**	C8A	Complement C8 Alpha Chain
**ENSG00000160712.13**	IL6R	Interlukin 6 Receptor
**ENSG00000161011.20**	SQSTM1	Sequestosome 1
**ENSG00000164171.11**	ITGA2	Integrin subunit alpha 2
**ENSG00000164251.5**	F2RL1	Coagulation Factor II (thrombin) receptor-like 1
**ENSG00000164946.19**	FREM1	FRAS1 (Fraser extracellular matrix complex subunit 1) related extracellular matrix 1
**ENSG00000165272.16**	AQP3	Aquaporin 3 (Gill blood group)
**ENSG00000166508.17**	MCM7	Minichromosome maintenance complex component 7
**ENSG00000185813.10**	PCYT2	Proteini Phosphate cytidylyltransferase 2, ethanolamine
**ENSG00000187134.14**	AKR1C1	Aldo-keto reductase family 1 member C1
**ENSG00000196208.14**	GREB1	Growth regulating estrogen receptor binding 1
**ENSG00000274588.2**	DGKK	Diacylglycerol kinase kappa

Table S2: The biological processes of differentially expressed genes when Hep G2 cells were treated with **1**.

GO_term_ID	GO_term_def	Genes
**GO:0048856**	anatomical structure development	AKR1C1|ANGPTL8|AQP3|CCN1|CTSH|DHRS2|F2RL1|FREM1|GLUD1|HMOX1|HOXD1|IL6R|ITGA2
**GO:0006950**	response to stress	AQP3|C8A|CCN1|DGKK|DHRS2|F2RL1|GABARAPL1|HMOX1|IL6R|ITGA2|MCM7|SQSTM1
**GO:0007165**	signal transduction	AKAP12|ANGPTL8|CTSH|DGKK|F2RL1|HMOX1|IL6R|ITGA2|SQSTM1
**GO:0002376**	Immune system process	C8A|CTSH|DHRS2|F2RL1|HMOX1|IL6R|PODXL2|SQSTM1
**GO:0030154**	cell differentiation	AKR1C1|ANGPTL8|CCN1|DHRS2|F2RL1|HOXD1|ITGA2|SQSTM1
**GO:0006810**	transport	AKAP12|AKR1C1|AQP3|ASGR1|CTSH|F2RL1|SLC13A5|SQSTM1
**GO:0006629**	lipid metabolic process	ABHD4|AKR1C1|ANGPTL8|DGKK|DHRS2|HADH|PCYT2
**GO:0042592**	homeostatic process	ABHD4|AKR1C1|ANGPTL8|AQP3|CTSH|F2RL1|HMOX1
**GO:0009056**	catabolic process	ABHD4|CTSH|GABARAPL1|GLUD1|HADH|HMOX1|SQSTM1
**GO:0022607**	cellular component assembly	AKR1C1|GABARAPL1|HMOX1|ITGA2|MCM7|SQSTM1
**GO:0040011**	locomotion	CCN1|F2RL1|IL6R|ITGA2|PODXL2
**GO:0008219**	cell death	CCN1|CTSH|HMOX1|IL6R|SQSTM1
**GO:0008283**	cell population proliferation	ANGPTL8|F2RL1|HMOX1|ITGA2|MCM7
**GO:0048870**	cell motility	CCN1|F2RL1|IL6R|ITGA2|PODXL2
**GO:0006464**	cellular protein modification process	ASGR1|CCN1|CTSH|SQSTM1
**GO:0065003**	protein-containing complex assembly	AKR1C1|HMOX1|MCM7|SQSTM1
**GO:0007155**	cell adhesion	CCN1|FREM1|ITGA2|PODXL2
**GO_term_ID**	GO_term_def	Genes
**GO:0009058**	biosynthetic process	ASGR1|GLUD1|MCM7|PCYT2
**GO:0044281**	small molecule metabolic process	AKR1C1|GLUD1|HADH
**GO:0003013**	circulatory system process	AKAP12|F2RL1|HMOX1

Table S2 cont'd: The biological processes of differentially expressed genes when Hep G2 cells were treated with 1.

GO_term_ID	GO_term_def	Genes
**GO:0016192**	vesicle-mediated transport	ASGR1|CTSH|SQSTM1
**GO:0009790**	embryo development	CCN1|HOXD1|ITGA2
**GO:0034641**	cellular nitrogen compound metabolic process	CTSH|HMOX1|MCM7
**GO:0048646**	anatomical structure formation involved in morphogenesis	CCN1|HMOX1|ITGA2
**GO:0030198**	extracellular matrix organization	CCN1|ITGA2
**GO:0055085**	transmembrane transport	AQP3|SLC13A5
**GO:0050877**	nervous system process	HOXD1|ITGA2
**GO:0015031**	protein transport	AKAP12|F2RL1
**GO:0034330**	cell junction organization	F2RL1|ITGA2
**GO:0044403**	symbiotic process	ASGR1|ITGA2
**GO:0051186**	cofactor metabolic process	AKR1C1|HMOX1
**GO:0000003**	reproduction	CCN1|ITGA2
**GO:0006914**	developmental maturation	GABARAPL1|SQSTM1
**GO:0007005**	mitochondrion organization	GABARAPL1|SQSTM1
**GO:0051604**	protein maturation	CTSH
**GO:0006605**	protein targeting	AKAP12
**GO:0000278**	mitotic cell cycle	MCM7
**GO:0007049**	cell cycle	MCM7
**GO:0019748**	secondary metabolic process	AKR1C1
**GO:0021700**	developmental maturation	ANGPTL8
**GO:0006259**	DNA metabolic process	MCM7
**GO:0006520**	cellular amino acid metabolic process	GLUD1
**GO:0051276**	chromosome organization	MCM7

Table S3: The molecular Function of differentially expressed genes when Hep G2 cells were treated with 1.

GO_term_ID	GO_term_def	P	P_FDR_adj	Genes
GO:0030335	positive regulation of cell migration	2.15E-06	9.66E-03	AKAP12|CCN1|F2RL1|IL6R|ITGA2
GO:2000147	positive regulation of cell motility	2.66E-06	9.66E-03	AKAP12|CCN1|F2RL1|IL6R|ITGA2
GO:0051272	positive regulation of cellular component movement	3.06E-06	9.66E-03	AKAP12|CCN1|F2RL1|IL6R|ITGA2
GO:0040017	positive regulation of locomotion	3.51E-06	9.66E-03	AKAP12|CCN1|F2RL1|IL6R|ITGA2
GO:0010562	positive regulation of phosphorus metabolic process	5.50E-06	1.01E-02	AKAP12|CCN1|F2RL1|IL6R|ITGA2|SQSTM1
GO:0045937	positive regulation of phosphate metabolic process	5.50E-06	1.01E-02	AKAP12|CCN1|F2RL1|IL6R|ITGA2|SQSTM1
GO:1905155	positive regulation of membrane invagination	1.79E-05	2.47E-02	F2RL1|ITGA2
GO:0060100	positive regulation of phagocytosis, engulfment	1.79E-05	2.47E-02	F2RL1|ITGA2
GO:0060099	regulation of phagocytosis, engulfment	2.41E-05	2.61E-02	F2RL1|ITGA2
GO:1905153	regulation of membrane invagination	2.75E-05	2.61E-02	F2RL1|ITGA2
GO:0030334	regulation of cell migration	2.80E-05	2.61E-02	AKAP12|CCN1|F2RL1|IL6R|ITGA2
GO:0009605	response to external stimulus	3.00E-05	2.61E-02	AKAP12|AQP3|CCN1|F2RL1|GABARAPL1|IL6R |ITGA2
GO:0002687	positive regulation of leukocyte migration	3.08E-05	2.61E-02	F2RL1|IL6R|ITGA2
GO:0050921	positive regulation of chemotaxis	3.61E-05	2.78E-02	F2RL1|IL6R|ITGA2
GO:2000145	regulation of cell motility	3.91E-05	2.78E-02	AKAP12|CCN1|F2RL1|IL6R|ITGA2
GO:0032270	positive regulation of cellular protein metabolic process	4.04E-05	2.78E-02	AKAP12|CCN1|F2RL1|IL6R|ITGA2|SQSTM1
GO:0040012	regulation of locomotion	5.70E-05	3.49E-02	AKAP12|CCN1|F2RL1|IL6R|ITGA2

Table S3 Cont'd: The molecular Function of differentially expressed genes when Hep G2 cells were treated with 1.

GO_term_ID	GO_term_def	P	P_FDR_adj	Genes
GO:0051247	positive regulation of protein metabolic process	5.86E-05	3.49E-02	AKAP12|CCN1|F2RL1|IL6R|ITGA2|SQSTM1
GO:0051270	regulation of cellular component movement	6.02E-05	3.49E-02	AKAP12|CCN1|F2RL1|IL6R|ITGA2
GO:0001934	positive regulation of protein phosphorylation	6.51E-05	3.49E-02	AKAP12|CCN1|F2RL1|IL6R|SQSTM1
GO:0050927	positive regulation of positive chemotaxis	6.86E-05	3.49E-02	F2RL1|ITGA2
GO:0050926	regulation of positive chemotaxis	7.43E-05	3.49E-02	F2RL1|ITGA2
GO:0019220	regulation of phosphate metabolic process	7.70E-05	3.49E-02	AKAP12|CCN1|F2RL1|IL6R|ITGA2|SQSTM1
GO:0051174	regulation of phosphorus metabolic process	7.75E-05	3.49E-02	AKAP12|CCN1|F2RL1|IL6R|ITGA2|SQSTM1
GO:0042327	positive regulation of phosphorylation	8.27E-05	3.49E-02	AKAP12|CCN1|F2RL1|IL6R|SQSTM1
GO:0010647	positive regulation of cell communication	8.34E-05	3.49E-02	AKAP12|CCN1|F2RL1|IL6R|ITGA2|SQSTM1
GO:0023056	positive regulation of signaling	8.56E-05	3.49E-02	AKAP12|CCN1|F2RL1|IL6R|ITGA2|SQSTM1
GO:0002685	regulation of leukocyte migration	1.04E-04	4.11E-02	F2RL1|IL6R|ITGA2

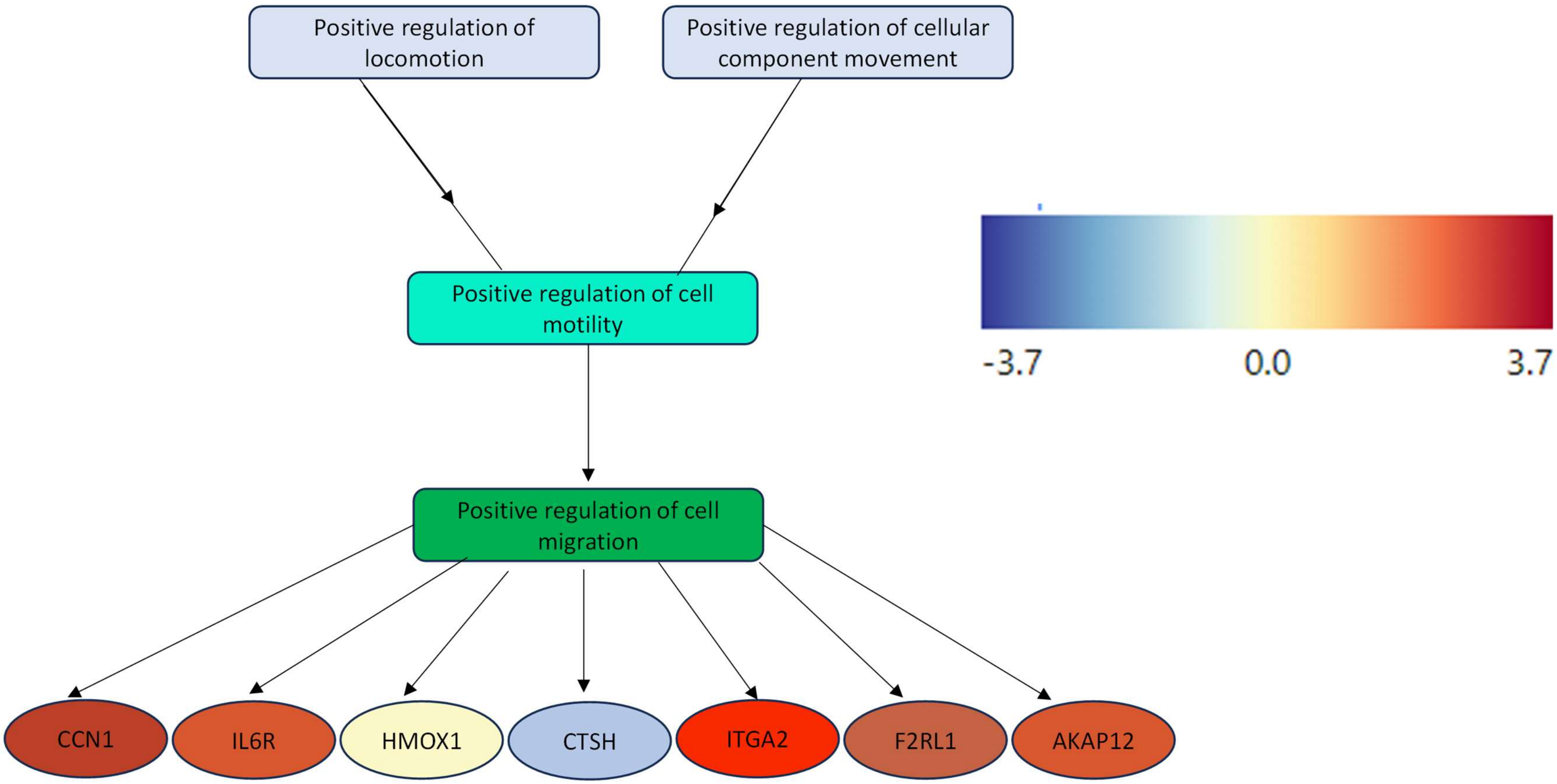

Figure S9: The Enriched pathway linked to the differentially expressed genes in cell treated with 1 across time point.

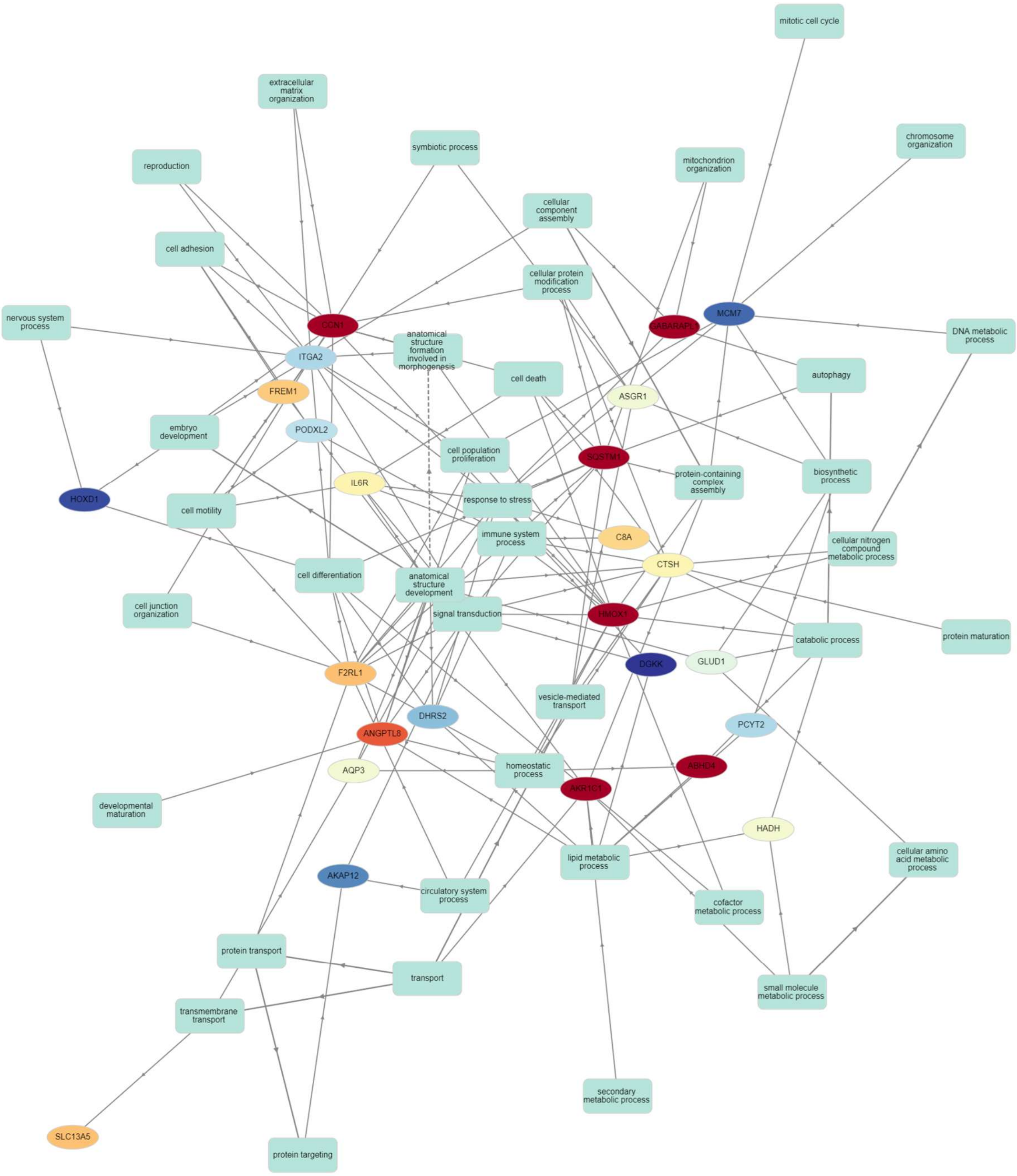

Figure S10: The interconnected pathway linked to the differentially expressed genes in cells treated with **1** across time points.

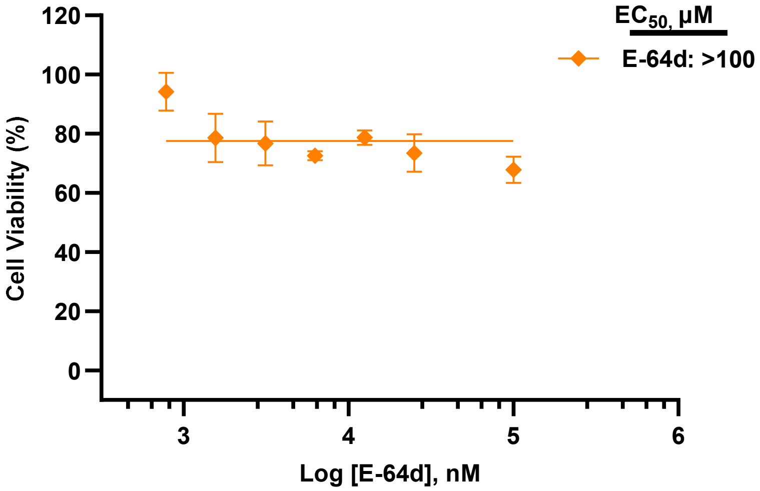

Figure S11: Antiproliferative activity of **E-64d** on hepatocellular carcinoma cells. Hep G2, 50,000 cells/well, were treated with **E-64d for** 72 h, and the cell viability was quantified as stated in the method section.
